# Assessing the multitargeted antidiabetic potential of three pomegranate peel‐specific metabolites: An in silico and pharmacokinetics study

**DOI:** 10.1002/fsn3.3644

**Published:** 2023-09-13

**Authors:** Hina Gull, Aqsa Ikram, Anees Ahmed Khalil, Zahoor Ahmed, Arash Nemat

**Affiliations:** ^1^ Faculty of Sciences, Institute of Molecular Biology and Biotechnology The University of Lahore Lahore Pakistan; ^2^ Faculty of Allied Health Sciences, University Institute of Diet and Nutritional Sciences The University of Lahore Lahore Pakistan; ^3^ School of Food and Biological Engineering Jiangsu University Zhenjiang China; ^4^ Department of Microbiology Kabul University of Medical Sciences Kabul Afghanistan

**Keywords:** antidiabetic, bioactive compounds, pharmacokinetic profiling, pomegranate peel, target proteins

## Abstract

Diabetes is a chronic metabolic disorder that occurs due to impaired secretion of insulin, insulin resistance, or both. Recent studies show that the antidiabetic drugs used to control hyperglycemic levels are associated with undesirable adverse effects. Therefore, developing a safe and effective medicine with antidiabetic potential is needed. In this context, in silico studies are considered a rapid, effectual, and cost‐effective method in drug discovery procedures. It is evident from the literature that plant‐based natural components have shown promising outcomes in drug development to alleviate various diseases and hence have diversified the screening of potential antidiabetic agents. Purposely, in the present study, an in silico approach was performed on three *Punica granatum* peel metabolites (punicalin, punicalagin, and ellagic acid). All these three compounds were docked against nine protein targets involved in glucose metabolism (GFAT, PTP1β, PPAR‐ᵞ, TKIR, RBP4, α‐amylase, α‐glucosidase, GCK, and AQP‐2). These three pomegranate‐specific compounds demonstrated significant interactions with GFAT, PTP1β, PPAR‐ᵞ, TKIR, RBP4, α‐amylase, α‐glucosidase, GCK, and AQP‐2 protein targets. Specifically, punicalin, punicalagin, and ellagic acid revealed significant binding scores (−9.2, −9.3, −8.1, −9.1, −8.5, −11.3, −9.2, −9.5, −10.1 kcal/mol; −10, −9.9, −8.5, −8.9, −10.4, −9.0, −10.2, −9.4, −9.0 kcal/mol; and −8.1, −8.0, −8.0, −6.8, −8.7, −7.8, −8.3, −8.1, −8.1 kcal/mol, respectively), with nine protein targets mentioned above. Hence, punicalin, punicalagin, and ellagic acid can be promising candidates in drug discovery to manage diabetes. Furthermore, in vivo and clinical trials must be conducted to validate the outcomes of the current study.

## Graphical abstract

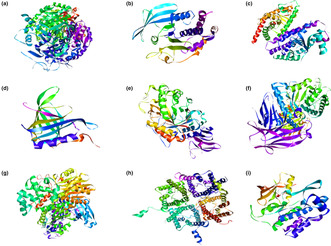

## 1 INTRODUCTION

Diabetes mellitus (DM) is a metabolic disorder characterized by a shortage of insulin production, insulin resistance, or both. The pathophysiology of type 2 diabetes mellitus (T2DM), a chronic condition with increasing global concern, involves elevated blood glucose levels and disruptions in the metabolism of carbohydrates, lipids, and proteins (Nazaruk & Borzym‐Kluczyk, 2015). The 2022 diabetic research report shows that more than 357 million people of all ages have diabetes, which will rise to 783 million by 2045 (Sun et al., 2022). One of the main types of diabetes is type 2 diabetes mellitus (T2DM), in which the body cells cannot respond to insulin, thus increasing blood sugar levels (Turkoski, 2006). T2DM is a metabolic disorder and a leading cause of cardiovascular disease (Goyal & Jialal, 2018; Shah et al., 2021). Some of the approaches to treat T2DM include regular exercise and change in lifestyle, which could be followed with medications such as thiazolidine derivatives, metformin, or sulfonylureas as a second line of treatment. However, these medications have limitations due to their potential side effects (gastrointestinal intolerance, nausea, hypoglycemia, headache, and dizziness) and lack of effectiveness in some cases (Chaudhury et al., 2017; Dujic et al., 2016). Thus, there is a need for more effective treatments for T2DM. In the management of type 2 diabetes mellitus (T2DM), targeting specific proteins implicated in the disease's development, such as glutamine: fructose‐6‐phosphate amidotransferase (GFAT), protein tyrosine phosphatase 1β (PTP1‐β), RBP‐4 (retinol binding protein‐4), α‐amylase, and α‐glucosidase, has shown promise (Murphy & Holder, 2000; Sun et al., 2017; Vyas et al., 2013). Inhibition of GFAT reduces the glycemic levels in the blood as it is the rate‐limiting enzyme of the hexosamine biosynthetic pathway (HBP), which is involved in developing insulin resistance and diabetic complications (Din et al., 2021). PTP1β is an integral membrane receptor and acts as a negative insulin‐signaling pathway regulator by hydrolyzing the phosphor‐tyrosine on the insulin receptor. Inhibition of protein tyrosine phosphatase 1β (PTP1β) restores normal blood glucose levels (Sun et al., 2016; Zhang & Zhang, 2007). Previous research has indicated that increased levels of circulating retinol binding protein‐4 (RBP4) are associated with insulin resistance, metabolic syndrome, impaired glucose tolerance, and T2DM (Kwanbunjan et al., 2018; Li et al., 2018; Pandey et al., 2015; Sun et al., 2014). Consequently, inhibiting RBP‐4 offers a preventive measure against the development of T2DM (Berry & Noy, 2017; Kwanbunjan et al., 2018). PPAR‐γ regulates glucose levels in peripheral tissues, especially in adipose tissue, thus these receptors act as molecular targets of drugs, such as the thiazolidinediones, which improve insulin sensitivity and glucose metabolism (Gurnell, 2003; Lu et al., 2011). α‐amylase and α‐glucosidase digest the carbohydrates and increase the postprandial glucose level, and inhibiting the activity of these two enzymes can control postprandial glucose levels and glycemic control in diabetic subjects (Poovitha & Parani, 2016). The glucose‐phosphorylating enzyme “glucokinase” was identified as an ideal drug target for developing antidiabetic medicines because it has an exceptionally high impact on glucose homeostasis because of its glucose sensor role in pancreatic β‐cells and as a rate‐controlling enzyme for hepatic glucose clearance and glycogen synthesis, both processes that are impaired in type 2 diabetes (Matschinsky, 2009). AQP2 mutation leads to nephrogenic diabetes insipidus (NDI), characterized by polyuria, polydipsia, and hypernatremia (Li et al., 2021).

New pharmaceutics can be developed by studying the interaction of plant constituents and targeted proteins. It will lead researchers to discover novel drugs from natural products. In recent times, there have been a growing interest in the utilization of computer‐aided drug discovery (CADD) methods due to their ability to address challenges related to scale, time, and cost encountered by traditional experimental approaches (Shaker et al., 2021; Tang et al., 2006). The field of CADD encompasses several computational processes, such as identifying potential drug targets, conducting virtual screening of extensive chemical libraries to identify promising drug candidates, optimizing specific constituents, and performing in silico evaluations to assess their potent toxicity (Brogi et al., 2020). Following the computational processes, candidate compounds identified through CADD undergo validation through in vitro and/or in vivo experiments. Therefore, implementing CADD techniques can effectively decrease the number of chemical compounds requiring conventional experimental evaluation, thereby enhancing the success rate by eliminating less effective and toxic candidates from consideration (Gupta et al., 2013; Segall & Barber, 2014). Computational research methods have significantly accelerated and made the novel drug discovery process more cost‐effective. In the last decade, advancements in predicting the ADMET properties of pharmaceutical compounds have led to improved characterization of these compounds (Butina et al., 2002; Rajalakshmi et al., 2021).

Pomegranate (*Punica granatum* Linn) is a fruit‐bearing deciduous shrub that belongs to the family “Punicaceae” (Johanningsmeier & Harris, 2011), typically in a season in the Southern Hemisphere from March to May and in the Northern Hemisphere from September to February (Sinha et al., 2012). Since ancient times, pomegranate has been used for medicinal purposes in China, Ayurveda, and Islamic and Persian regions (Ge et al., 2021). Various parts of the pomegranate tree, bark, fruit, root, rind, fruit juice, seed, seed oil, stem, leaf, peel, and flower are known to have antioxidant, nitric oxide promoter, anticholesterolemic, antihypertensive, antiviral, antibacterial, anticandidal, and anticancer properties (Middha et al., 2012; Sun, 2012). Several bioactive compounds have been isolated from pomegranate peel. The phytochemical study of pomegranate peel has resulted in isolating secondary metabolites of medicinal importance such as proanthocyanidins, ellagitannins, alkaloids, flavonoids, coumarins, pigments, fatty acids, sterols, and proteins (Fahmy & Farag, 2022; Ranjha et al., 2021). In traditional medicine, various parts of plants, such as peels, roots, stems, leaves, and fruits, are used as remedies (Iqbal et al., 2022; Manzoor et al., 2021; Tan et al., 2021). However, there is limited validated information available from previous phytochemical studies. In this study, we investigated the potential antidiabetic effects of the specific metabolites found in pomegranate peel, ellagic acid, punicalagin, and punicalin using in silico methods. The present study aimed to evaluate the multitargeted potential punicalin, punicalagin, and ellagic acid in *Punica granatum* peel against target protein related to glucose metabolism and diabetes mellitus.

## 2 MATERIALS AND METHODS

The current study was divided into three main parts: 1. Selection of proteins involved in the pathogenesis of diabetes; 2. Selection of phytochemicals reported in *Punica granatum* peels as ligand molecules; and 3. Docking analysis of screened proteins and phytochemicals. ADMET profiling was done to get safe and effective drug discovery eventually.

### 2.1 Target selection and preparation

**FIGURE 1:**
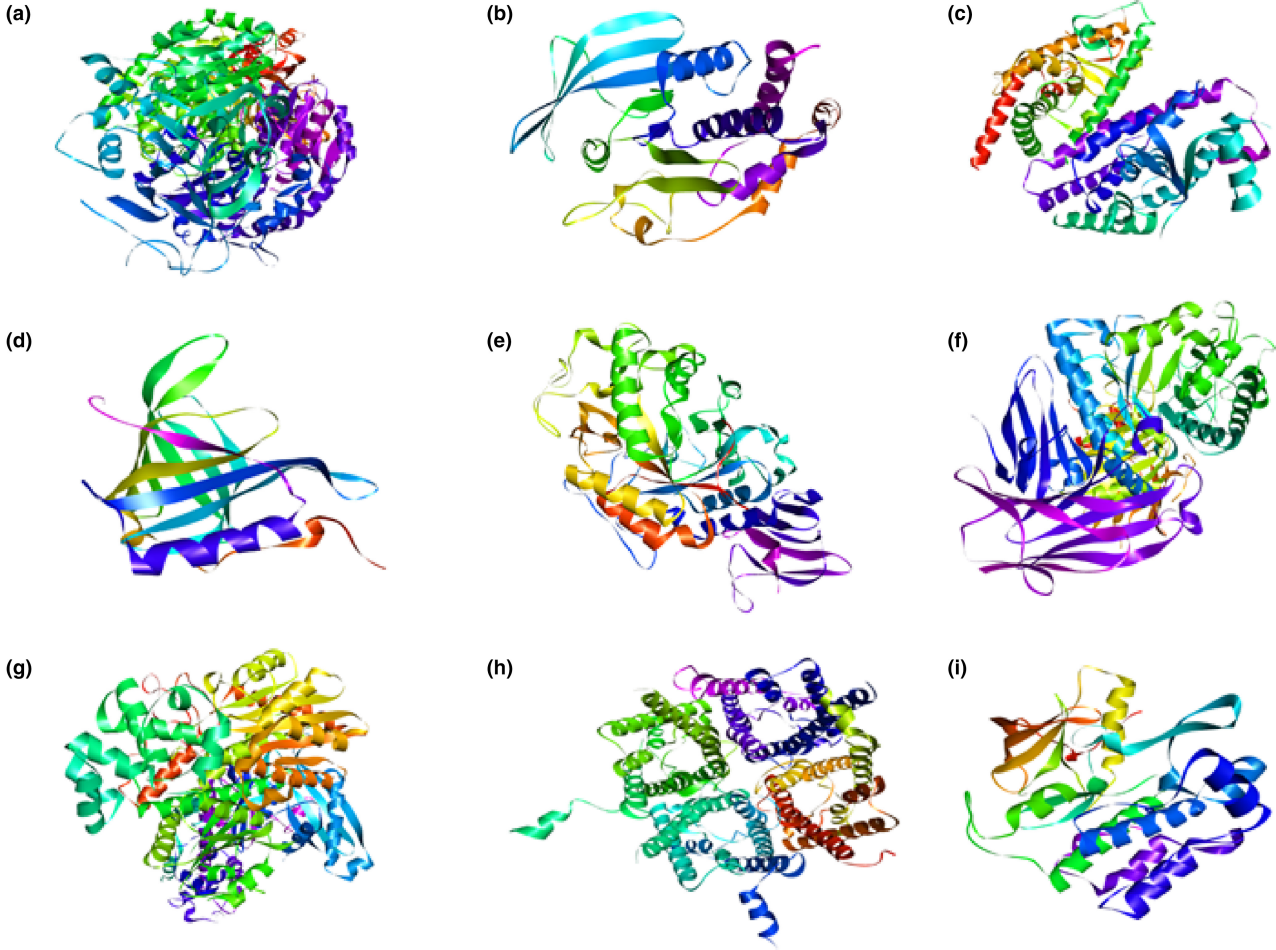
3D structures of proteins included in the present study: (a) GFAT, (b) PTP1β, (c) PPAR‐γ, (d) RBP‐4, (e) α‐amylase, (f) α‐glucosidase, (g) GCK, (h) AQP‐2, and (i) TK‐IR.

Strict criteria were followed to select the target proteins causing diabetes mellitus. These proteins were selected through a literature survey based on “the most cited” in literature and were experimentally validated both in vitro and in vivo. The chosen nine target proteins with their PDB IDs were as follows: glutamine fructose‐6‐phosphate amidotransferase (GFAT) (PDB ID: 6ZMJ), protein tyrosine phosphatase 1 beta (PTP1β) (PDB ID: 3EB1), peroxisome proliferator‐activated receptors gamma (PPAR‐ᵞ) (PDB ID: 4YT1), tyrosine kinase insulin receptors (RTKs) (PDB ID: 1IRK), retinol binding protein 4 (RBP4) (PDB ID: 1BRP), alpha‐amylase (α‐amylase) (PDB ID: 2QMK), alpha‐glucosidase (α‐glucosidase) (PDB ID: 5KZW), glucokinase (GCK) (PDB ID: 4LC9), and aquaporin 2 (AQP2) (PDB ID: 4NEF) as shown in Figure 1. The targeted proteins' three‐dimensional (3D) structures were retrieved from RCSB Protein Data Bank (https://www.rcsb.org/) in .pdb format. Protein structures were refined using PyMol software (https://pymol.org/edu/index.php) and Chimera 1.15 (https://www.cgl.ucsf.edu/chimera/download.html). It includes the removal of solvents, deleting all ligand molecules, and adding polar hydrogen, which was energy minimized.

### 2.2 Ligand selection and preparation

**FIGURE 2:**
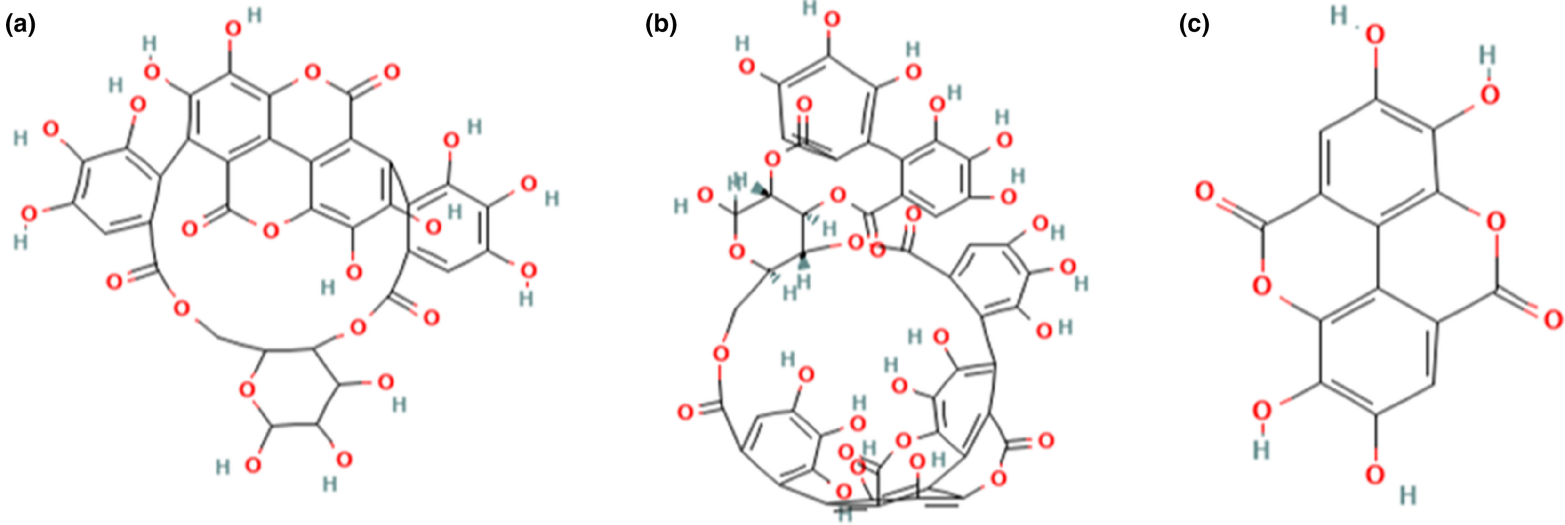
2D structures of phytochemical compounds included in the present study: (a) punicalin, (b) punicalagin, and (c) ellagic acid.

The bioactive metabolites of pomegranate peel were used as ligand molecules screened via literature mining. Selected compounds of pomegranate peel were punicalin, punicalagin and ellagic acid as these compounds are present in abundant quantities in pomegranate peel as a comparison to other fruits and are also reported in various studies (Adams et al., 2006; Chen et al., 2013; García et al., 2021; Gonzalez‐Castillo et al., 2021; Lansky, 2006; Lu et al., 2008; Sabraoui et al., 2020; Wang et al., 2013). The phytochemical compounds' 3D or 2D chemical structures were retrieved from Pubchem source (https://pubchem.ncbi.nlm.nih.gov/) in .sdf format (Figure 2) and converted into PDB format in PyMol.

### 2.3 Active sites prediction

The binding sites of proteins were determined via the coach server and 3D ligandscout online tool using PDB files as input. Predicted participating amino acids in the pockets are: GFAT: Arg407, Glu393, Gln386, Tyr547, Asp642, His597, Glu390, Asn408, Glu390, Asp642, Gln986, Tyr547, Arg407, Arg502, Ser498, Ala263, Met311, Gln422, Glu469, Asn448, Gly467, Pro468. For PTP1‐ β: Gln262, Gly183, Arg221, Glu115, Lys120, Asp48, Tyr46, Tyr46, Asp48, Arg221, Trp179, Glu115, Ser216, Asp181, Thr263, Lys116, Phe182, Tyr46, Lys120, Gly220, Gln266, Trp179, Gly183, Asp181, Arg221, Lys116, Ser216. For RBP‐4: Gln262, Gly183, Arg221, Glu115, Lys120, Asp48, Tyr46, Tyr46, Asp48, Arg221, Trp179, Glu115, Ser216, Asp181, Thr263, Lys116, Phe182, Tyr46, Lys120, Gly220, Gln266, Trp179, Gly183, Asp181, Arg221, Lys116, Ser216. For AQP‐2: Arg152, Glu232, Leu4, His61, Arg11, Ile62, Arg85, Gly60, Glu232, His61, Asp150, Gly154, His61, Val56, Arg153, Gly158, Pro160, Glu155. For GCK: Lys51, Gly54, Ser310, Thr13, His303, Ala58, Gln304, Ser308, Asn246, Val244, Ser78, Thr82, Gln237, Arg478, Lys475, Thr471, and Asp413. For α‐amylase: Glu240, Lys200, Glu233, Leu162, Ser163, His305, Gly306, Tyr151, Asp290, Arg421, Asp402, Ser4, Asn5, Asn216, His215, Lys227, Pro228, Leu211, and Asp212. For α‐glucosidase: Arg275, Tyr543, Asp319, Asp91, Met122, Arg591, His432, Arg437, Asp508, Gln429, Pro511, Pro595, Arg608, Ser864, Arg594, Val867, and His717. For PPAR‐γ: Arg280, Ile341, Ser342, Arg288, Gln444, Arg443, Arg397, His323, Val446, Val450, Lys319, Gln444, Leu476, Arg397, Pro398, Asp396, and Arg443. For TK‐IR: Arg1131, His1130, Leu1170, Gly1152, Asp1150, Arg1131, Asp1132, Tyr1162, Asp1150, Glu1047, Asp1083, Phe1151, Met1153, and Asp1150.

### 2.4 Molecular docking

Docking analysis was done via PyRx, a Virtual Screening software for Computational Drug Discovery. It includes Autodock4, Autodock Vina, Autodock tools, and VTK (visualization tool kit). Gridbox coordinates and size were set according to the active pockets of the target protein. Polar hydrogen atoms were added to the pdb structures of the proteins so strong bonds could be produced with ligands wherever possible. The 3D structure of the protein molecule was converted in pdbqt format via open babel in PyRx. 2D structures will be shifted to the 3D scene via “open babel” in Pyrx. Energy minimization of ligand molecules was done via PyRx. The ligand was converted into pdbqt format for docking analysis, as PyRx accepts only .pdbqt format for docking analysis. Pyrx was run to get the binding affinity measurements.

### 2.5 Visualization

Interactions with the highest binding energy scores or the best‐docked pose were retrieved for further analysis. Finally, 3D and 2D interactions between proteins and ligands were studied and optimized in BIOVIA Discovery Studio 2021.

### 2.6 Absorption, distribution, metabolism and excretion, and toxicity profile/toxicological profiling or pharmacokinetics

Pharmacokinetic properties include absorption, distribution, metabolism and excretion, and toxicity profile/toxicological (ADMET) endpoints. The pharmacokinetic properties of phytocompounds were evaluated using SwissADME. It was used to investigate the ADMET qualities of the phytocompounds from the *Punica granatum* dried peel powder extract. The toxicological endpoints (hepatotoxicity, carcinogenicity, immunotoxicity, and mutagenicity) and the level of toxicity (LD50, mg/Kg) of the phytocompounds were determined using ProTox‐II server. Pharmacokinetics properties (GI absorption, BBB permeation, P‐gp substrate, cytochrome‐P enzymes inhibition, and skin permeation [log Kp]) are the key criteria for predicting the absorption and distribution of drugs within the body.

## 3 RESULTS

The 2D and 3D interactions of the docking results were obtained by importing our results into the discovery studio visualizer, allowing us to discover important interactions between the ligands and the receptor‐binding site.

### 3.1 Molecular docking

#### 3.1.1 Protein glutamine: fructose‐6‐phosphate amidotransferase with ligands punicalin, punicalagin, and ellagic acid

**FIGURE 3:**
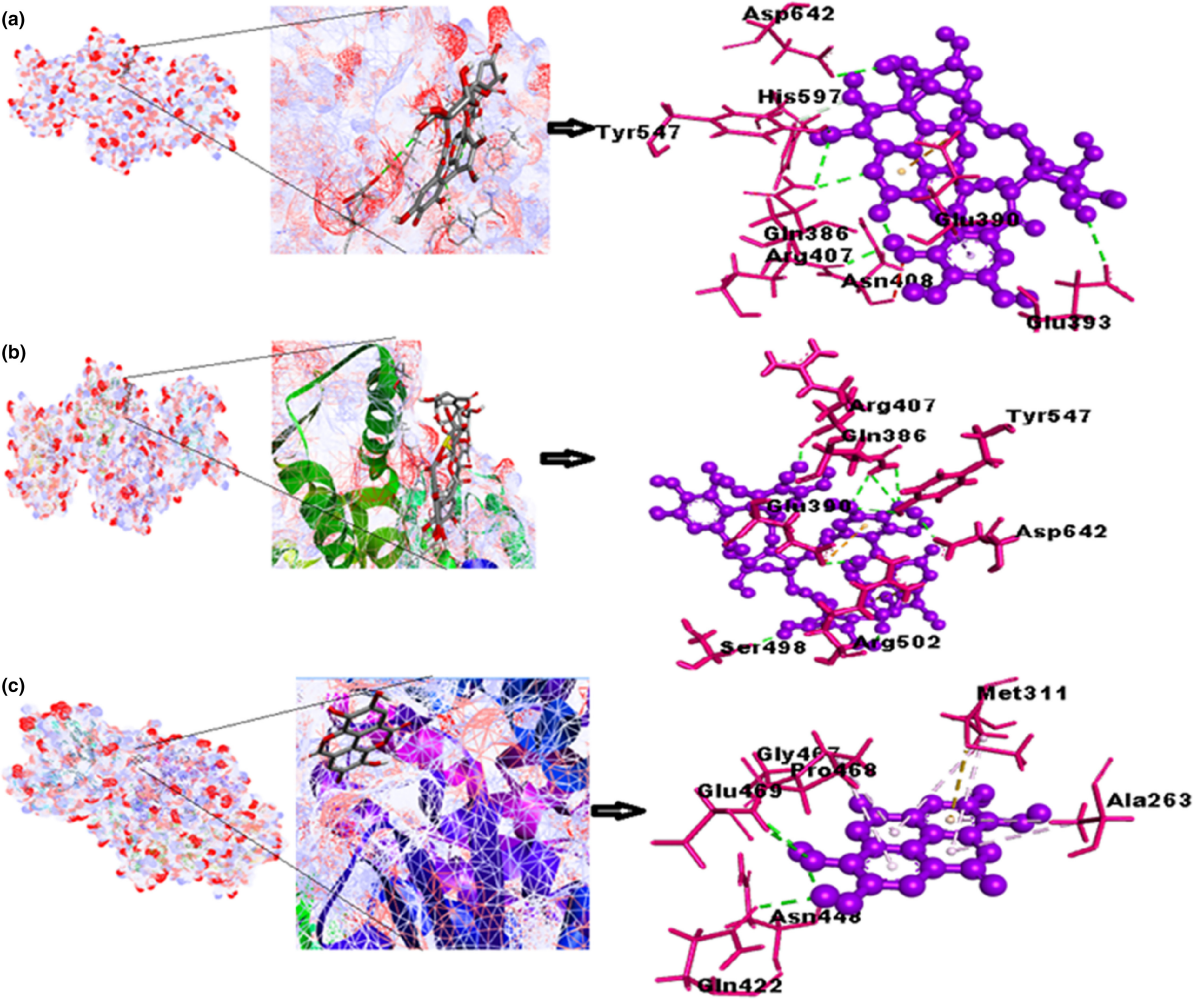
(a) Best‐docked poses of GFAT with punicalin ligand (mesh figure with ribbon form), 3D views of punicalin with the surrounding amino acids of GFAT; (b) best‐docked poses of GFAT with punicalagin ligand (mesh figure with ribbon form), 3D view of punicalagin with surrounding amino acids of GFAT; (c) best‐docked poses of GFAT with ellagic acid ligand (mesh figure with ribbon form), 3D view of ellagic acid with the surrounding amino acids of GFAT (Figure S1): 2D interactions of GFAT with punicalin, punicalagin, and ellagic acid.

The bioactive compound punicalin binds with GFAT satisfactorily with a free binding energy of −9.2 kcal/mol. It demonstrates attachment with six H‐bonds among six different amino acids, as depicted in Figure 3 and Figure S1; additional examination reveals the existence of a π‐cation interaction with Glu390. Additionally, the complex displayed hydrophobic contacts carried out by the aliphatic amino acid Asn408. The binding ability of punicalagin with GFAT is good, that is, −10 kcal/mol. Eighteen hydrogen bonds are identified with the 10 different amino acids, of which 7 are the conventional H‐bonding, whereas 8 are carbon H‐bonding. Two pi‐cation interactions are present with amino acids Arg502 and Glu390. The binding energy of ellagic acid with GFAT is −8 kcal/mol. It formed 5 hydrogen bonds with 5 different amino acids. Further study shows a π‐sulfur interaction with Met311, and π‐alkyl interactions with Ala263, Met311, and Pro468 amino acids.

#### 3.1.2 Protein peroxisome proliferator‐activated receptors gamma with ligands punicalin, punicalagin, and ellagic acid

**FIGURE 4:**
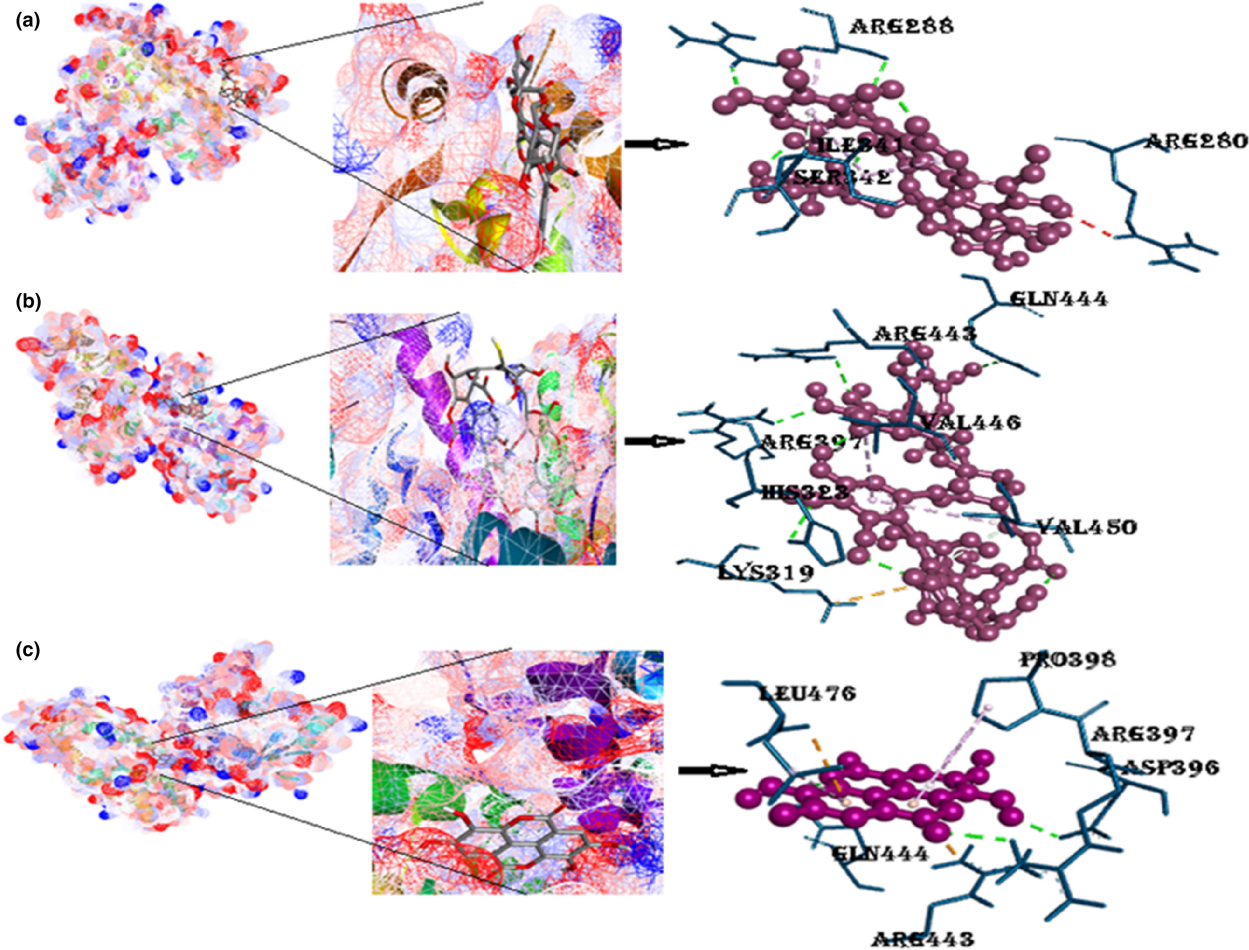
(a) Docked complexes of PPAR‐γ with punicalin ligand (mesh figure with ribbon‐structured protein), closeup view of protein–ligand interaction, punicalin with encompassing amino acids of 4YT1; (b) docked complexes of PPAR‐γ with punicalagin ligand (mesh figure with ribbon‐structured protein), closeup view of protein–ligand interaction, punicalagin with encompassing amino acids of 4YT1; (c) docked complexes of PPAR‐γ with ellagic acid (mesh figure with ribbon‐structured protein), closeup view of protein–ligand interaction, ellagic acid with encompassing amino acids of 4YT1 (Figure S2): 2D interactions of PPAR‐γ with punicalin, punicalagin, and ellagic acid.

Peroxisome proliferator‐activated receptors gamma docks excellently with the punicalin with the free binding energy of −8.1 kcal/mol. Three hydrogen bonds were formed between the PPAR‐ᵞ and punicalin; two illustrated conventional hydrogen bonding with the same amino acid Arg288 with different benzene rings (Figure 4 and Figure S2). In addition, a pair of pi‐alkyl bonds are formed with the same amino acid Ile341, and the other is Arg288. Arg280 forms an unfavorable donor–donor bond. The binding energy of the punicalagin with protein was stated as −8.5 kcal/mol as shown in Table 2. There were four conventional hydrogen bonds present with the three different amino acids, a couple of amino acids Arg (Arg397 and Arg443), His 323, and Gln444. The configurational energy of the punicalagin was reduced by the occurrence of π‐cation interaction (Lys319), stable with 30° angle of π‐alkyl interaction with Val (450, 446) on the nearby ring of the compound. Three H‐bonds are formed between PPAR‐ᵞ and ellagic acid. Arg443 and Leu476 formed pi‐cation and pi‐anion bonds, respectively, with the drug, while Pro398 formed an π‐alkyl bond with the cyclohexane backbone of the ellagic acid.

#### 3.1.3 Protein tyrosine phosphatase 1β with ligands punicalin, punicalagin, and ellagic acid

**FIGURE 5:**
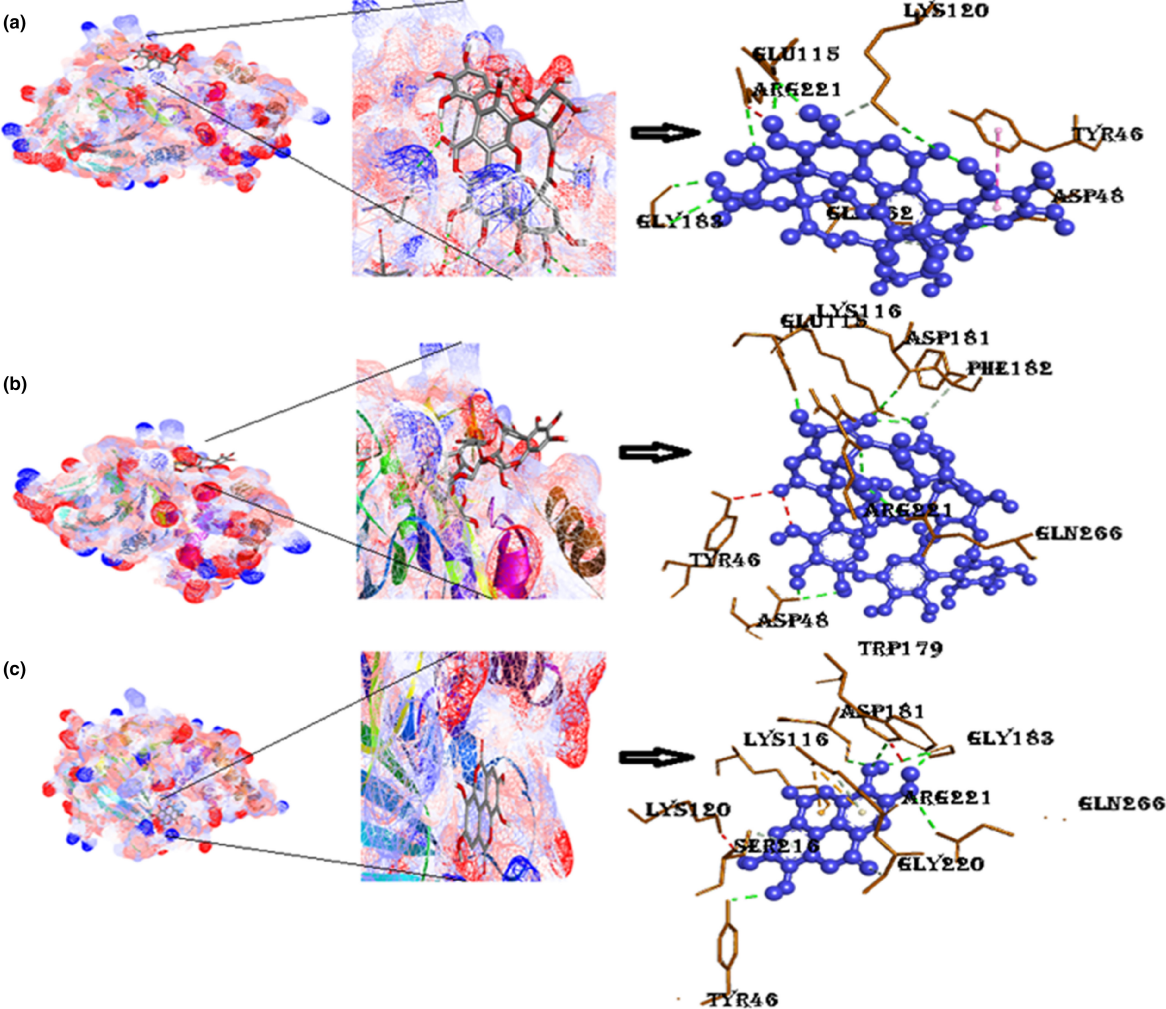
The docking model of known PTP1β (3EB1); (a) best‐docked model of punicalin with 3EB1, their closeup image, their 3D interactions; (b) best‐docked model of punicalagin with 3EB1, closeup view of ligand–protein interaction, 3D ligand interactions; (c) docking model of ellagic acid in the active site of 3EB1, their closeup image, 3D 3EB1 interactions with ellagic acid. (Figure S3): 2D interactions of PTP1β with punicalin, punicalagin, and ellagic acid.

A docked structure of PTP1β with punicalin shown in Figure 5 indicates that binding was feasible with a free binding energy (−9.3 kcal/mol) as majority of the interaction affinities were of hydrogen bond types with different amino acids, which were Gln262, Gly183, Arg221, Glu115, Lys120, and Asp148. Thus, resulting in a net negative value. The complex stability is associated with additional π‐sigma interactions linked with π‐alkyl (Ile881, Ile963, Ile831, Ala805, and Met804), π‐sulfur (Met953), and π‐cation (Lys833) interactions. The binding energy of the punicalagin with PTP1β comes out to be −9.9 kcal/mol with nine conventional hydrogen bonds, one carbon‐hydrogen bond, and two unfavorable donor‐donor interactions (Trp179, Arg221). The docked result of PTP1β with ellagic acid is displayed in Figure 5 and Figure S3, which indicates that the drug has eight hydrogen bond interactions with seven amino acids. Pi‐cation interactions were with two amino acids, Arg221 and Lys116, that no doubt resulted in a solid, cohesive environment, thus, stabilizing the formed complex. Other significant hydrophobic interactions are Trp179 and Lys120.

#### 3.1.4 Protein retinol binding protein‐4 with ligands punicalin, punicalagin, and ellagic acid

**FIGURE 6:**
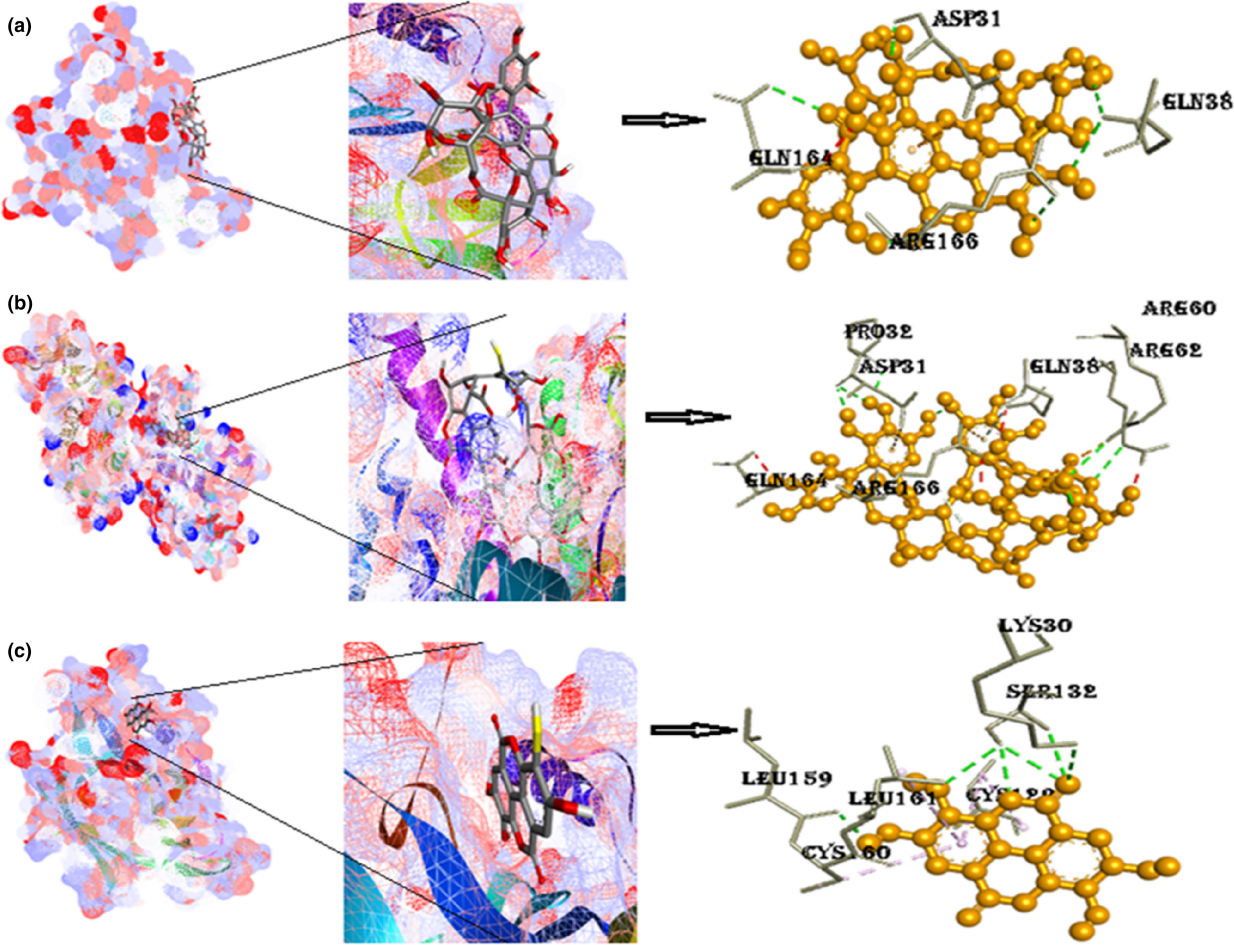
(a) Docked postures of RBP4 with punicalin, their closeup view, 3D views of RBP4 with neighboring amino acids of 1BRP; (b) docked postures of RBP4 with punicalagin, their closeup view, 3D view of RBP4 with neighboring amino acids of 1BRP; (c) docked postures of RBP4 with ellagic acid, their closeup view, 3D view of RBP4 with neighboring amino acids of 1BRP (Figure S4): 2D interactions of RBP4 with punicalin, punicalagin, and ellagic acid.

The docked complex of RBP4 with punicalin represented in Figure 6 depicts a negative free energy of binding (−9.3 kcal/mol) which includes interactions of hydrogen bonding with amino acids (Gln38, Arg166, Asp31, Gln164) with large number of hydrophobic interactions, therefore, providing the overall negative value. The complex via acceptor–acceptor interaction (Gln164) achieves further stability. A complex of protein RBP4 with punicalagin shows the binding energy −8.9 kcal/mol having four hydrogen bonds, one Pi‐cation (Lys319), and two Pi‐alkyl bonds (Val446 and Val450) with the central point of the same ring, thus increases in the stability of the protein complex molecule (Figure 6 and Figure S4 **)**. The binding energy of RBP4 with ellagic acid compound is −6.8 kcal/mol. All hydrogen bonds are conventional hydrogen bonds with amino acids Lys30, Leu159, and Ser132. Other Pi‐alkyl interactions are with Cys129, Cys160, and Leu161 and amino acids.

#### 3.1.5 Protein glucokinase with ligands punicalin, punicalagin, and ellagic acid

**FIGURE 7:**
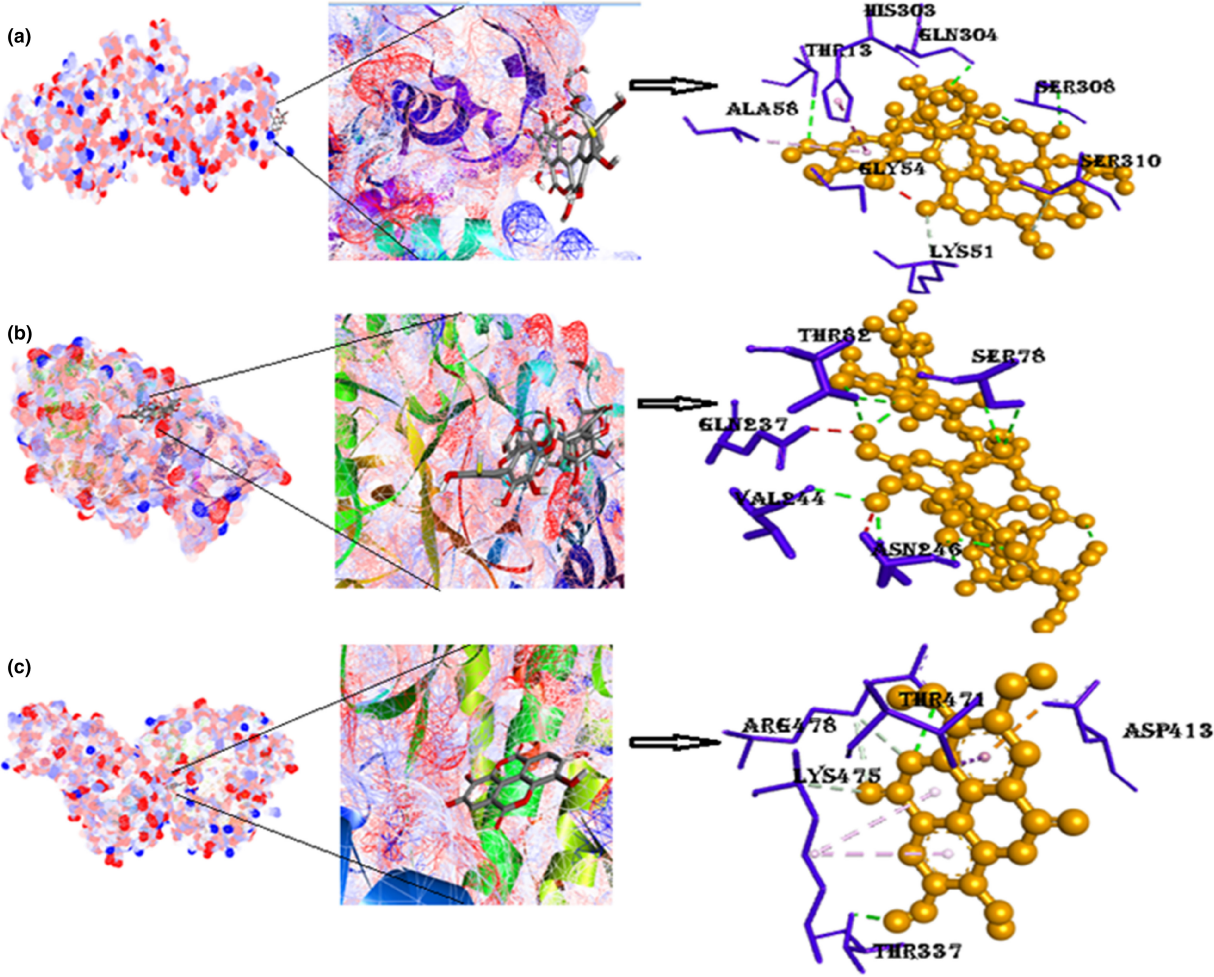
(a) Docked position of GCK with punicalin, 3D views with bordering amino acids of 4LC9; (b) docked position of glucokinase with punicalagin, 3D view of interaction type of punicalagin with bordering amino acids of 4LC9; (c) docked position of GCK with ellagic acid, closeup image of this docked complex, 3D view of interaction type of ellagic acid with bordering amino acids of 4LC9. (Figure S5): 2D interactions of GCK with punicalin, punicalagin, and ellagic acid.

Glucose‐metabolizing enzyme glucokinase with punicalin is demonstrated in Figure 7; the binding energy is given in Table 2, to be −9.5 kcal/mol. The results shown in Figure 7 indicate that the drug has two conventional hydrogen bonds and two carbon–hydrogen bond interactions with four amino acids, as listed in Table 2. Other significant interactions, such as π‐alkyl and π‐π‐T‐shaped interactions, are with amino acids Ala58 and His303, respectively. Binding energy of 4FA6 with punicalagin is −9 kcal/mol. Five hydrogen bonds were found in the complex formation between punicalagin and 4FA6 with amino acids Ser, Asn, and Asp, and Pi‐cation interaction is with Arg421. Docked structure of GCK with ellagic acid presented in Figure 7 and Figure S5 is of negative binding energy (−7.8 kcal/mol). This structure has four hydrogen bonds with three different amino acids shown in the above figure. Other noticeable interactions are Pi‐sigma with Thr471 and Pi‐anion interaction with Asp413 amino acid.

#### 3.1.6 Protein AQP‐2 with ligands punicalin, punicalagin, and ellagic acid

**FIGURE 8:**
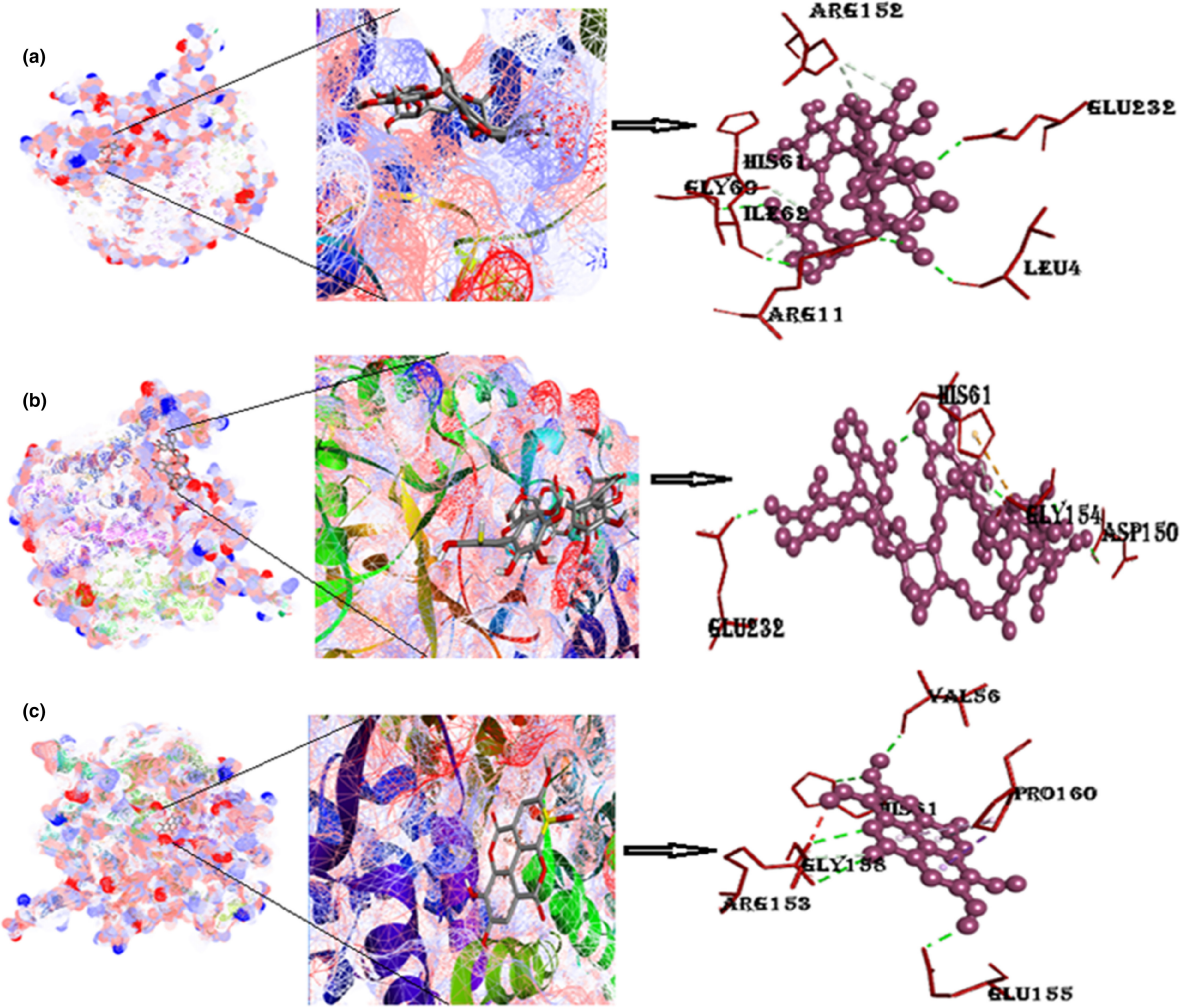
(a) Docked model of AQP‐2 with punicalin, 3D views with encircling amino acids of 4NEF; (b) docked model of punicalagin with 4NEF, their closeup view, 3D view of interaction type of punicalagin with encircling amino acids of 4NEF; (c) docked model of AQP‐2 with ellagic acid, their closeup image, 3D view of interaction type of ellagic acid with encircling amino acids of 4NEF (Figure S6): 2D interactions of AQP‐2 with punicalin, punicalagin, and ellagic acid.

The binding energy of AQP‐2 with the punicalin is −10.1 kcal/mol, 10 hydrogen bonds attained this interaction, and no other type of interaction is found there. The binding energy of AQP‐2 with punicalagin comes out to be −10.4 kcal/mol—the highest binding affinity among all other docked structures. It consists of hydrogen bonding with amino acids Glu232, Asp150, and His61. Pi‐anion interaction with His61 and donor–donor interaction with Gly154 give the complex a compact shape. Complex molecule of AQP‐2 with ellagic acid contains seven flexible bonds on the drug and π‐sigma interaction (Pro160), introducing stabilizing charges responsible for intercalating the drug within the (4NEF). Two Pi‐alkyl bonds with the same amino acid on the adjacent rings and one π‐donor–donor bond with Arg153 in the binding region of the receptor Figure 8 and Figure S6.

#### 3.1.7 Protein TK‐IR with ligands punicalin, punicalagin, and ellagic acid

**FIGURE 9:**
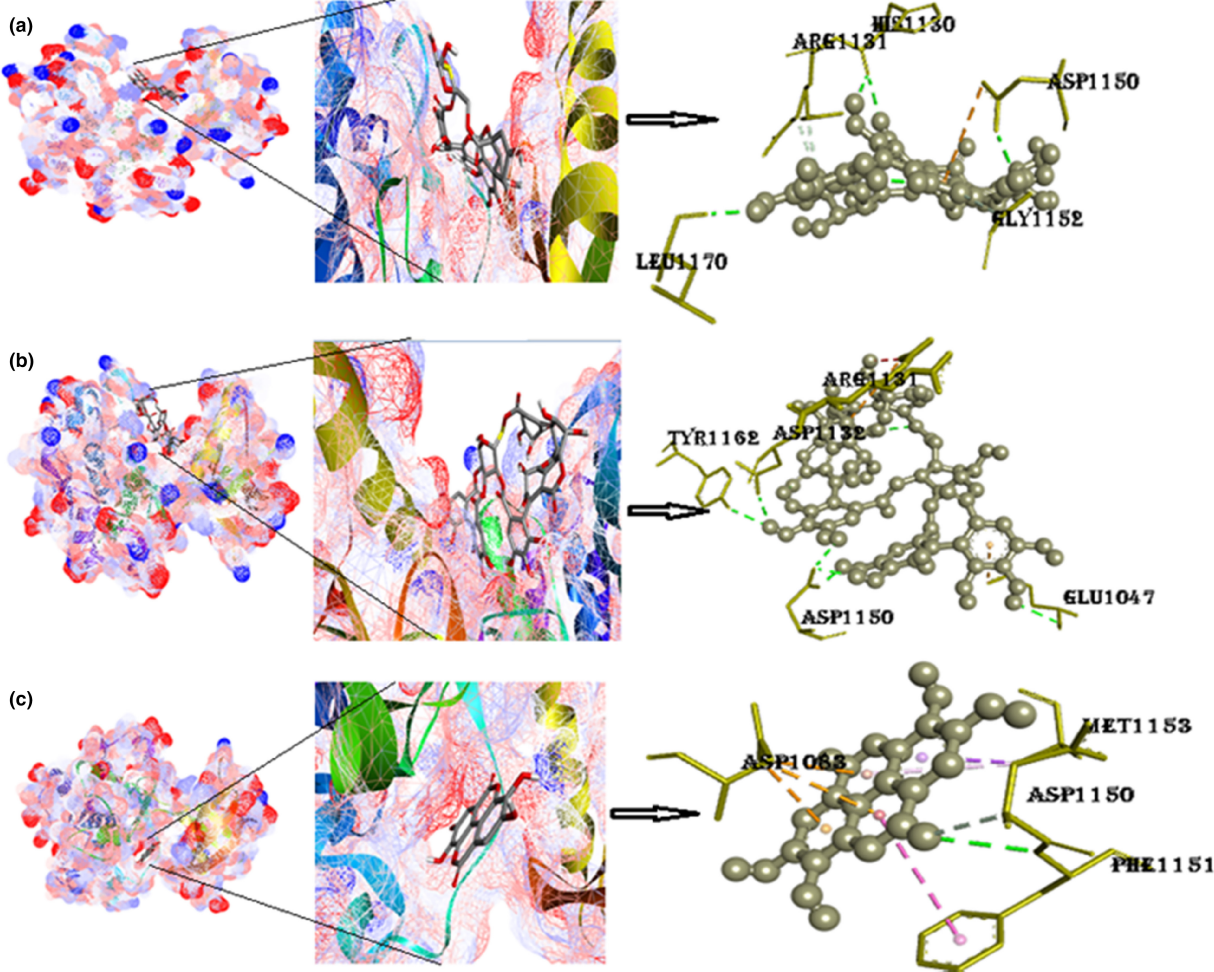
(a) Docked postures of TK‐IR with punicalin ligand (mesh figure with ribbon‐structured protein), closeup view of protein–ligand interaction, punicalin with enclosing amino acids of 1IRK; (b) docked postures of TK‐IR with punicalagin ligand (mesh figure with ribbon‐structured protein), closeup view of protein–ligand interaction, punicalagin with enclosing amino acids of 1IRK; (c) Docked postures of TK‐IR with ellagic acid ligand (mesh figure with ribbon‐structured protein), closeup view of protein–ligand interaction, ellagic acid with enclosing amino acids of 1IRK; (Figure S7): 2D interactions of TK‐IR with punicalin, punicalagin, and ellagic acid.

The binding energy of TK‐IR with punicalin is −8.5 kcal/mol. Five H‐bonds were determined in the complex of punicalin with TK‐IR with five amino acids (Table 2). The Pi‐anion interaction is with Asp1150. The binding energy of punicalagin with 1IRK is −9.0 kcal/mol. There are presently six hydrogen atoms with amino acids in Figure 9 and Figure S7. Other significant interactions are with Pi‐anion (Arg1131) balanced with an attractive charge of amino acid Glu1047. The binding energy of ellagic acid with TKIR is −8.1 kcal/mol. Two hydrogen bonds are present in this complex. The numerous π‐interactions, such as the π‐π interaction with Phe1151, π‐alkyl interaction with Met1153, and finally, the Pi‐Sigma interaction with Met1153, resulting from the hetero‐aromatic ring are thought to be the cause of ellagic acid's exceptional stability in the binding site. The numerous π‐sigma interactions (π‐alkyl and π‐sulfur), the majority of which involve charge transfer, aid in interpolating the drug in the receptor's binding site.

#### 3.1.8 Protein α‐amylase with ligands punicalin, punicalagin, and ellagic acid

**FIGURE 10:**
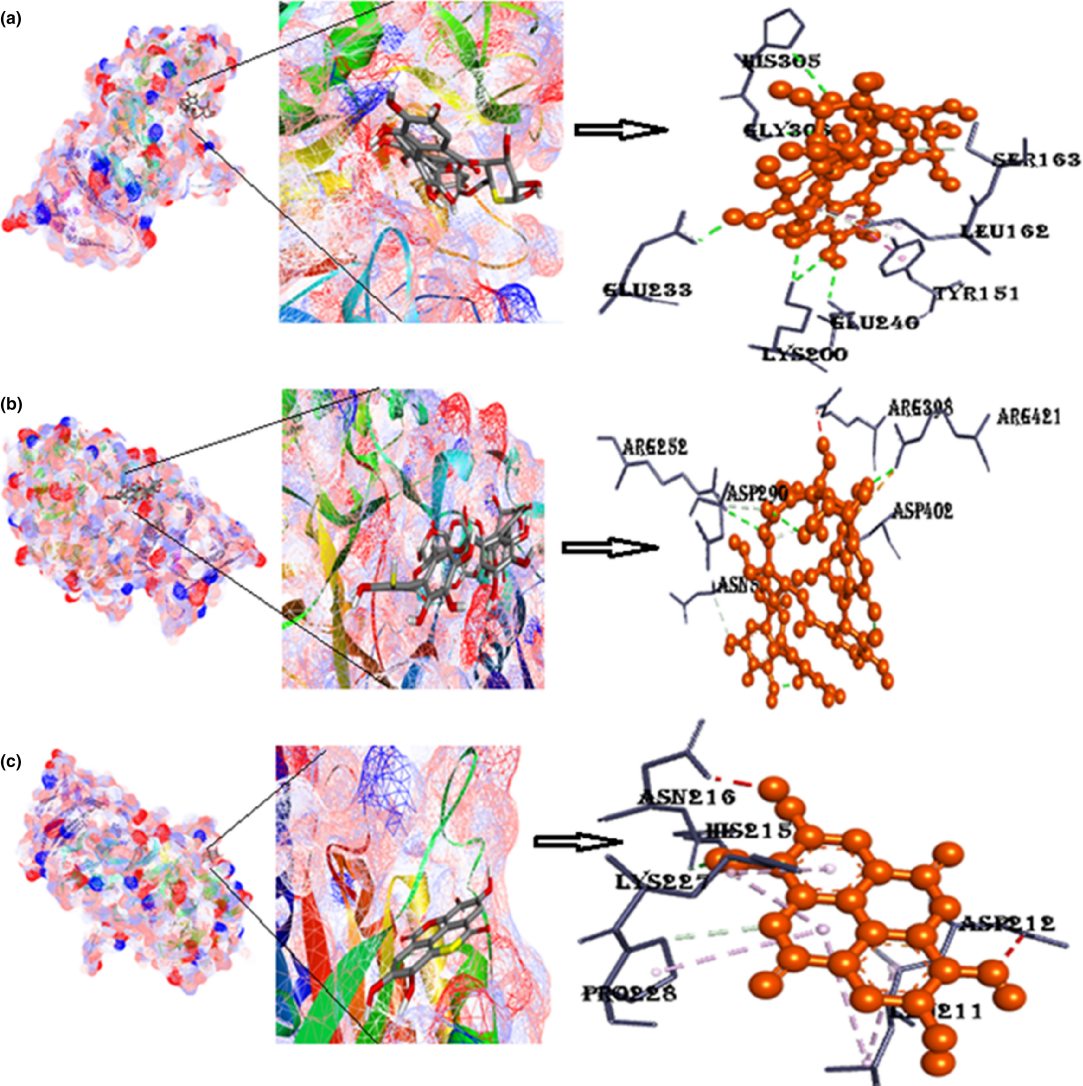
(a) Docked poses of α‐amylase with punicalin ligand (mesh figure with ribbon‐structured protein), closeup view of protein‐ligand interaction, punicalin with the nearby amino acids of 2QMK; (b) docked complexes of α‐amylase with punicalagin ligand (mesh figure with ribbon‐structured protein), closeup view of protein–ligand interaction, punicalagin with the nearby amino acids of 2QMK; (c) docked complexes of α‐amylase with ellagic acid ligand (mesh figure with ribbon‐structured protein), closeup view of protein–ligand interaction, ellagic acid with the nearby amino acids of 2QMK; (Figure S8): 2D interactions of α‐amylase with punicalin, punicalagin, and ellagic acid.

The binding energy of α‐amylase with punicalin is −11.3 kcal/mol. Punicalin forms hydrogen bonds with two amino acids (His215 and Pro228) that are listed in Table 2. Despite being a small protein, α‐amylase was found to have a high binding affinity, making it a better drug than 90% of all the other drugs in this study. The binding energy of α‐amylase with punicalagin is −10.2 kcal/mol. Amino acids involved in hydrogen bonding are Asn5, Arg421, Asp290, Asp402, and Ser4. Pi interactions included are the Pi‐cation Arg421. The binding energy of α‐amylase with ellagic acid is −8.3 kcal/mol. It consists of five Pi‐alkyl interactions with one hydrogen bonding, as shown in Figure 10 and Figure S8.

#### 3.1.9 Protein α‐glucosidase with ligands punicalin, punicalagin, and ellagic acid

**FIGURE 11:**
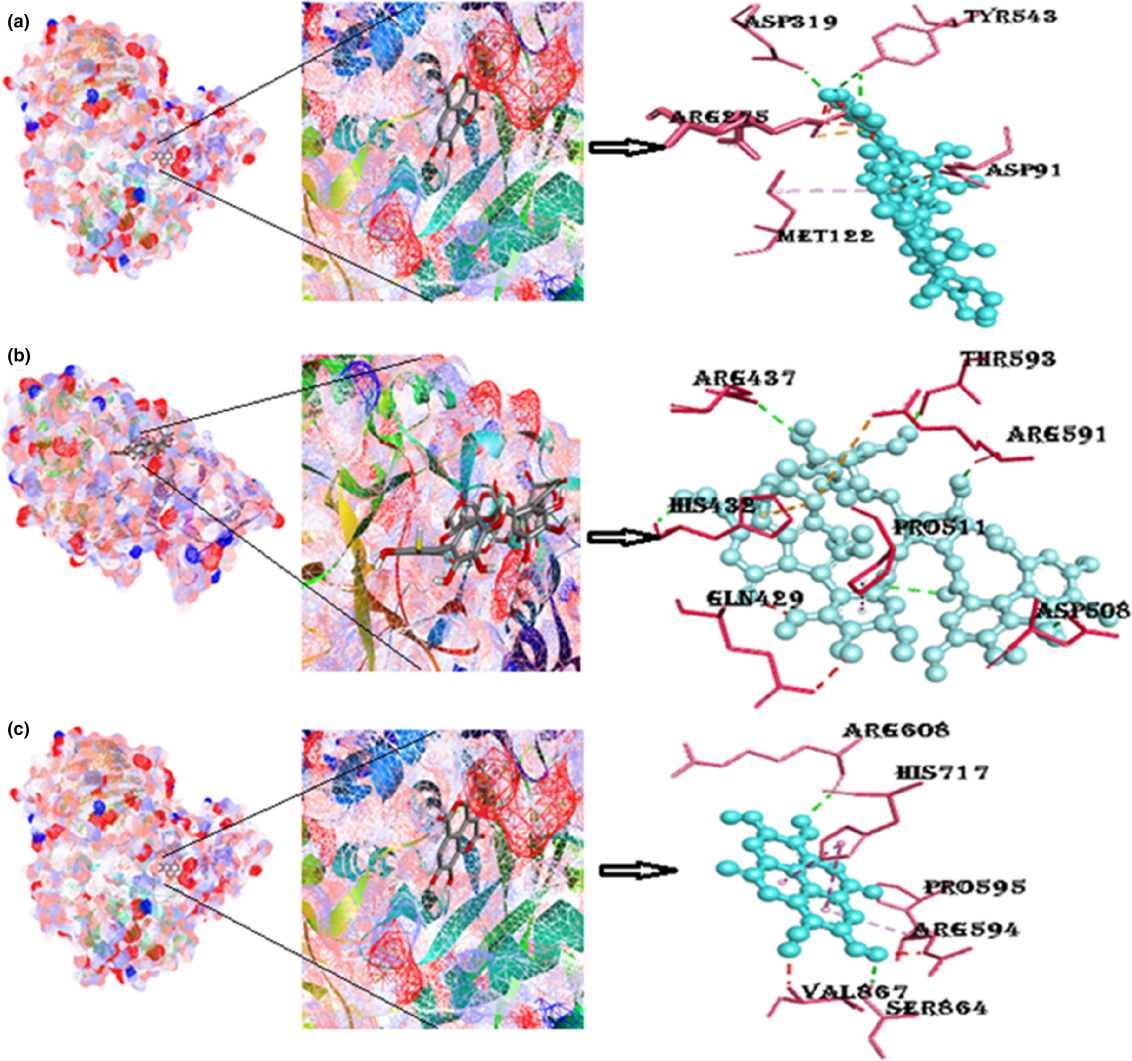
(a) Docked poses of α‐glucosidase with punicalin ligand (mesh figure with ribbon‐structured protein), closeup view of protein–ligand interaction, punicalin with neighboring amino acids of 5KZW; (b) docked structures of α‐glucosidase with punicalagin ligand (mesh figure with ribbon‐structured protein), closeup view of protein–ligand interaction, punicalagin with neighboring amino acids of 5KZW; (c) docked structures of α‐glucosidase with ellagic acid ligand (mesh figure with ribbon‐structured protein), closeup view of protein–ligand interaction, ellagic acid with neighboring amino acids of 5KZW (Figure S9): 2D interactions of α‐glucosidase with punicalin, punicalagin, and ellagic acid.

The binding energy of α‐glucosidase with punicalin is −9.2 kcal/mol. Two amino acids are involved in a conventional hydrogen bonding with punicalin (Asp319, Tyr543), which is reported in Table 1. The binding energy of α‐glucosidase with punicalagin is −9.4 kcal/mol. Amino acids involved in hydrogen bonding are Asn5, Arg421, Asp290, Asp402, and Ser4. Pi‐interactions included are the Pi‐cation Arg421. The binding energy of α‐glucosidase with ellagic acid comes out to be −9.4 kcal/mol. It consists of five Pi‐alkyl interactions with one hydrogen bonding, as shown in Figure 11 and Figure S9.

**TABLE 1:** Binding affinities, no. of hydrogen bonds, and participating amino acids of three ligands with nine selected proteins.

Serial No.	Ligand name	Protein name	Binding energy (kcal/Mol)	No. of H‐bonds	Participating amino acids
1	Punicalin	PPAR‐γ	−8.1	3	Arg, Ser
2		GFAT	−9.2	6	Asp, His, Tyr, Gln, Arg, Glu
3		PTP1‐β	−9.3	9	Gly, Gln, Pro, Arg, Trp, Asp
4		RBP‐4	−9.1	5	Gln, Asp, Arg, Gln
5		AQP‐2	−10.1	10	Glu, Arg, Leu, Arg, Ile, His, Gly
6		GCK	−9.5	6	Ser, Gln, Thr, Gly, Lys, Ser
7		α‐Amylase	−11.3	8	His, Thr, Glu, Lys
8		α‐Glucosidase	−9.2	3	Asn, Arg
9		TK‐IR	−8.5	6	His, Leu, Asp, Arg, Gly
10		PPAR‐γ	−8.5	4	Arg, Gln, His
11		GFAT	−10	7	Arg, Tyr, Gln, His, Asp, Glu, Ser, Pro, Met
12		PTP1‐β	−9.9	10	Asp, Gln, Arg, Lys, Asp, Phe, Glu
13	Punicalagin	RBP‐4	−8.9	4	His, Arg, Gln
14		AQP‐2	−10.4	3	Gly, Asp, His
15		GCK	−9.0	5	Ser, Arg, Asn, Asp
16		α‐Amylase	−10.2	9	Asp, Arg, Pro, Gly, Ser
17		α‐Glucosidase	−9.4	4	Asp, Arg, His
18		TK‐IR	−9.0	1	Arg
19	Ellagic acid	PPAR‐γ	−8.1	3	Gln, Asp, Arg
20		GFAT	−8.0	5	Gln, Glu, Asn, Gly, Pro
21		PTP1‐β	−8.0	8	Gly, Ser, Gln, Trp, Asp
22		RBP‐4	−6.8	5	Ser, Lys, Cys, Leu
23		AQP‐2	−8.7	6	His, Val, Arg, Gly
24		GCK	−7.8	3	Lys, Arg
25		α‐Amylase	−8.3	2	His, Pro
26		α‐Glucosidase	−8.3	3	Ser, Pro, Arg
27		TK‐IR	−8.1	2	Asp, Phe

### 3.2 Pharmacokinetics

Pharmacokinetics helps prescribers adjust dosage more accurately. Therefore, while studying the pharmacokinetics of the selected compounds, among the three compounds, punicalin was found to be nontoxic and displayed good gastrointestinal absorption with a skin permeability of −7.36 cm/s. The top‐scored compound from docking analysis MOL009272 unveiled a molecular weight of 782.55 Da and AlogP as 0.83, as shown in Table 2. Numerous compounds failed in clinical trials due to poor ADMET properties. Therefore, ADMET properties are considered important criteria to ensure the drug likeness of hit molecules. The molecular weight was a bit higher than the accepted rule, that is, 500 Da, but the usual source and all other parameters facilitate the development of the potential above‐hit molecule against diabetes, which can act as a guide for drug discovery. ADMET profiling of three bioactive compounds is shown in Table 3.

**TABLE 3:** Pharmacokinetics of punicalin, punicalagin, and ellagic acid.

Pharmacokinetic properties	Punicalin	Punicalagin	Ellagic acid
GI absorption	High	Low	High
BBB permeant	No	No	No
P‐gp substrate	No	Yes	No
CYP1A2 inhibitor	Yes	No	Yes
CYP2C19 inhibitor	No	No	No
CYP2C9 inhibitor	No	No	No
CYP2D6 inhibitor	No	No	No
CYP3A4 inhibitor	No	No	No
Log Kp (skin permeation; cm/s)	−7.36	−11.67	No
Bioavailability score	0.55	0.17	0.55
Hepatotoxicity	Inactive	Inactive	Active
Carcinogenicity	Inactive	Inactive	Inactive
Immunotoxicity	Active	Inactive	Active
Mutagenicity	Inactive	Inactive	Inactive
Cytotoxicity	Inactive	Inactive	Inactive
LD50 (mg/Kg)	5000	5000	1190
Toxicity class	5	5	4

**TABLE 2:** Other features of selected phytochemical compounds for drug discovery via TCMSP.

Serial No.	Molecule ID	Molecule name	Mol. Wt (Da)	OB (%)	DL	AlogP	Caco‐2	BBB	TPSA
1	MOL009272	Punicalin	782.55	16.37	0.03	0.83	−2.87	−3.53	385.24
2	MOL009146	Punicalagin	1084.75	18.17	0.00	3.00	−3.44	−4.46	518.76
3	MOL001002	Ellagic acid	302.20	43.06	0.43	1.48	−0.44	−1.41	141.34

Abbreviations: BBB, blood–brain barrier; DL, drug likeness; OB, oral bioavailability; TPSA, topological surface area.

## 4 DISCUSSION

This article discusses the potential of pomegranate peel‐specific compounds as a natural remedy for diabetes, a chronic metabolic disorder affecting millions worldwide. The study comprises the in silico analysis to investigate the interaction between pomegranate peel bioactive compounds (punicalin, punicalagin, and ellagic acid) and various proteins involved in glucose metabolism, including GFAT, PTP1‐β, PPAR‐ᵞ, RBP4, α‐amylase, α‐glucosidase, GCK, AQP‐2, and TKIR.

The study's results demonstrate the significant binding interactions of the pomegranate peel bioactive compounds with several proteins involved in glucose metabolism. Furthermore, their pharmacokinetics evaluation and ADMET profiling, hydrogen bonds involved in these interactions, interactive residues, and molecular features used for drug discovery were also investigated in this study. According to the results summarized in Table 1, binding affinities of punicalin, punicalagin, and ellagic acid to target proteins (GFAT, PTP1‐β, PPAR‐ᵞ, RBP4, α‐amylase, α‐glucosidase, GCK, AQP‐2, and TKIR) ranged from −6.8 to −11.3 kcal/mol, demonstrate the strong inhibitory effects. In addition, the compounds were found to inhibit the activity of α‐amylase and α‐glucosidase, enzymes responsible for the breakdown of carbohydrates into glucose. Punicalin, punicalagin, and ellagic acid are the most abundant phenolic compounds in various parts of pomegranate, specifically peels. These phenolic compounds possess antidiabetic potential through multiple pathways, including inhibition of digestive enzymes, reduction in oxidative stress, decreased inflammation, and reduction in protein glycation (Cardullo et al., 2020). These phenolic compounds may compete with enzymatic substrates and/or interact with the active site of an enzyme, therefore causing inhibition of enzymatic activity (Olvera‐Sandoval et al., 2022; Sancho & Pastore, 2012). Studies have shown that ellagitannins (punicalin and punicalagin) and their derivatives (ellagic acid) present in pomegranate peel extracts are responsible for the α‐amylase inhibitory potentials (Çam & İçyer, 2015; Karagecili et al., 2023).

Furthermore, the study found that pomegranate peel bioactive compounds positively affected GCK, AQP‐2, TKIR, and PPAR‐γ, all proteins involved in insulin signaling and glucose uptake. It suggests that pomegranate peel's bioactive compounds may benefit glucose metabolism and insulin sensitivity. From the structure of docking conformation, it has been analyzed that all ligands show good binding affinities with the target proteins of diabetes. The highest binding efficacy of α‐amylase with punicalin having binding energy (−11.3 kcal/mol) revealed that the ligand is tightly fixed in the pocket leading to the suppression of α‐amylase, thus, portrayed good interactions representing a useful, promising drug applicant. Overall, the antidiabetic effect of pomegranate peel compounds punicalin, punicalagin, and ellagic acid with proteins GFAT, PTP1β, PPAR‐γ, RTKs, RBP4, α‐amylase, α‐glucosidase, GCK, AQP‐2, and TKIR highlights the potential of these compounds as a natural treatment for diabetes and glucose metabolism disorders.

Various in silico studies have shown the effect of plant phenolics and flavonoids on several reported targets involved in diabetes. For instance, Balamurugan et al. (2012) revealed that γ‐sitosterol (sterol) isolated from *Lippia nodiflora* showed significant binding affinity (−7.49) with glucokinase. Glucokinase is exclusively found in liver and β‐cells of the pancreas and is critical in maintaining stable glucose levels in the body. In liver cells, phosphorylation of glucose by glucokinase promotes glucose uptake and metabolism by creating a gradient for glucose transport into the cells, thus controlling glucose disposal in the liver. In β‐cells, glucokinase is thought to play a role in the glucose‐sensing mechanism and regulate insulin release (Toye et al., 2004). During diabetes, total or partial insulin resistance leads to disruptions in carbohydrate metabolism, reducing glucokinase activity (Pari & Srinivasan, 2010), which results in decreased utilization of glucose and increased glucose production in the liver. According to Chandramohan et al. (2008), rats with diabetes treated with the active ingredient 3‐HMX from a plant showed increased glucokinase activity. The insulin receptor is a tyrosine protein kinase that activates upon insulin binding. Upon activation, it phosphorylates substrate proteins on multiple Tyr residues to transmit the insulin signal and carry out insulin's effects (Chandramohan et al., 2008). Studies on both activated and inactive insulin receptor tyrosine kinases have shown that in its activated state, the activation loop is tris‐phosphorylated and moves away from the active site. The phosphorylated tyrosines form hydrogen bonds with residues in the activation loop (De Meyts & Whittaker, 2002).

Recently, Kwon et al. (2022) chose branches of Morus alba for an in vitro assay and molecular docking analysis to examine the activity of two enzymes linked to diabetes mellitus and its complications. All tested compounds displayed more potent inhibition against α‐glucosidase compared to the positive control, particularly oxyresveratrol and kuwanon G. Of the compounds, kuwanon C has a prenyl component, demonstrated a more effective α‐glucosidase inhibitory effect compared to dihydromorin and norartocarpetin (Kwon et al., 2022). Earlier, Tadera et al. (2006) found that an increase in the number of hydroxyl (OH) groups and the presence of OH groups in flavonoids play a crucial role in inhibiting α‐glucosidase activity.

Similarly, punicalagin showed the most potent activity against PTP1β, with binding affinity −9.9 kcal/mol followed by punicalin and ellagic acid (Table 1). Nonpolar and hydrophobic components improved PTP1β inhibitory activity and cellular permeability (Tadera et al., 2006). Therefore, the inhibitory effects of punicalin, punicalagin, and ellagic acid are believed to be due to an increase in bioavailability caused by the presence of a carbonyl group and multiple aromatic rings. Selected pomegranate peel phytochemicals show pretty higher binding energies than bioactive compounds obtained from Okra (Ashraf et al., 2021; Lau et al., 2004), *Lippia nodiflora* (Balamurugan et al., 2012), banana flower (Ganugapati et al., 2012), *Morus alba* branches (white mulberry; Kwon et al., 2022), and neem (Jalil et al., 2013), with the selected proteins, which indicates pomegranate peel is better and more potent pharmaceutical agent than banana, okra, nodiflora fruit, white mulberry, and neem for the treatment of diabetes.

According to a study, punicalagin in pomegranate peel inhibited the activity of α‐glucosidase with an IC50 value of 82 μg/mL. Moreover, in silico analysis conducted in this study showed a binding affinity of −7.99 kcal/mol among punicalagin and α‐glucosidase. They believed punicalagin could be a potential candidate for developing antidiabetic functional food (Liu et al., 2022). Earlier, El Deeb et al. (2021) also reported the antidiabetic effect of ethyl acetate fraction of pomegranate rind in an experimental diabetic rat model, revealing its therapeutic potential for the management of hyperglycemia (El Deeb et al., 2021). Pomegranate peel powder and resultant extracts have been found as potential candidates for the management of diabetes. Pomegranate peel contains ellagitannins (ellagic acid, punicalagin, and punicalin) that help in reducing fasting blood glucose contents and are considered to be responsible for their antidiabetic properties. Methanolic extract of pomegranate peel powder possesses antidiabetic properties that may be due to the inhibition of α‐glucosidase activity (Arun et al., 2017). Similarly, Šavikin et al. (2018) revealed that ethyl acetate and aqueous fractions of pomegranate peel possess rich punicalin, ellagic acid, and punicalagin content. They reported that all the experimented fractions had significant α‐glucosidase and α‐amylase inhibitory activity with IC50 values ranging from 0.26 to 4.57 μg/mL and 23.6 to 284.3 μg/mL, respectively (Šavikin et al., 2018).

Based on their interactions and pharmacokinetics, these three ligands were the most promising candidates for antidiabetic agents. It has been found that these compounds exhibit varying pharmacokinetic profiles, with punicalin and ellagic acid having a more prolonged gut absorption time compared to punicalagin. Punicalin is nontoxic, whereas ellagic acid has more drug‐likeness properties. Punicalin and ellagic acid got a good molecular weight for becoming drug candidates, whereas ellagic acid got more AlogP value. Thus, punicalin has more potential and promising drug candidates among the three selected bioactive compounds than the others.

## 5 CONCLUSIONS

The present study sheds light on the effectiveness of polyphenols (ellagic acid, punicalagin, and punicalin) found in pomegranate peel against diabetic proteins GFAT, PTP1β, RBP‐4, α‐amylase, and α‐glucosidase. Results showed that punicalin, punicalagin, and ellagic acid play important roles in blood glucose regulation, which might be via activation of GCK, TK‐IR, and PPAR‐γ and correction of AQP‐2. In addition, the molecular docking and pharmacokinetics study show that the components of pomegranate peel (ellagic acid, punicalagin, and punicalin) are likely prospects for further drug discovery through in vitro studies. As they are part of a food product, they already have a confirmed safety profile, making them a desirable choice in treating diseases.

## AUTHOR CONTRIBUTIONS

**Hina Gull:** Conceptualization (equal); data curation (equal); formal analysis (equal); resources (equal); software (equal). **Aqsa Ikram:** Data curation (equal); formal analysis (equal); software (equal). **Anees Ahmed Khalil:** Conceptualization (equal); formal analysis (equal); writing – review and editing (equal). **Zahoor Ahmed:** Formal analysis (equal); writing – review and editing (equal). **Arash Nemat:** Formal analysis (equal); writing – original draft (equal).

## CONFLICT OF INTEREST STATEMENT

The authors declare no conflict of interest.

## Supporting information


Figures S1–S9

## Data Availability

The dataset supporting the conclusions of this article is included in the report. Additionally, 2D interactions are available as supplementary files.
