# Supplementary material for: Assessing the multitargeted antidiabetic potential of three pomegranate peel‐specific metabolites: An in silico and pharmacokinetics study
Source: Food Sci Nutr. 2023 Sep 13;11(11):7188–205. doi: 10.1002/fsn3.3644 (PMC10630828; doi:10.1002/fsn3.3644)
Supplement: Supplementary file 1 — Figures S1–S9 [file FSN3-11-7188-s001.docx]

Assessing the multi-targeted anti-diabetic potential of three pomegranate peel-specific metabolites: An in-silico and pharmacokinetics study

**Supplementary Figures**


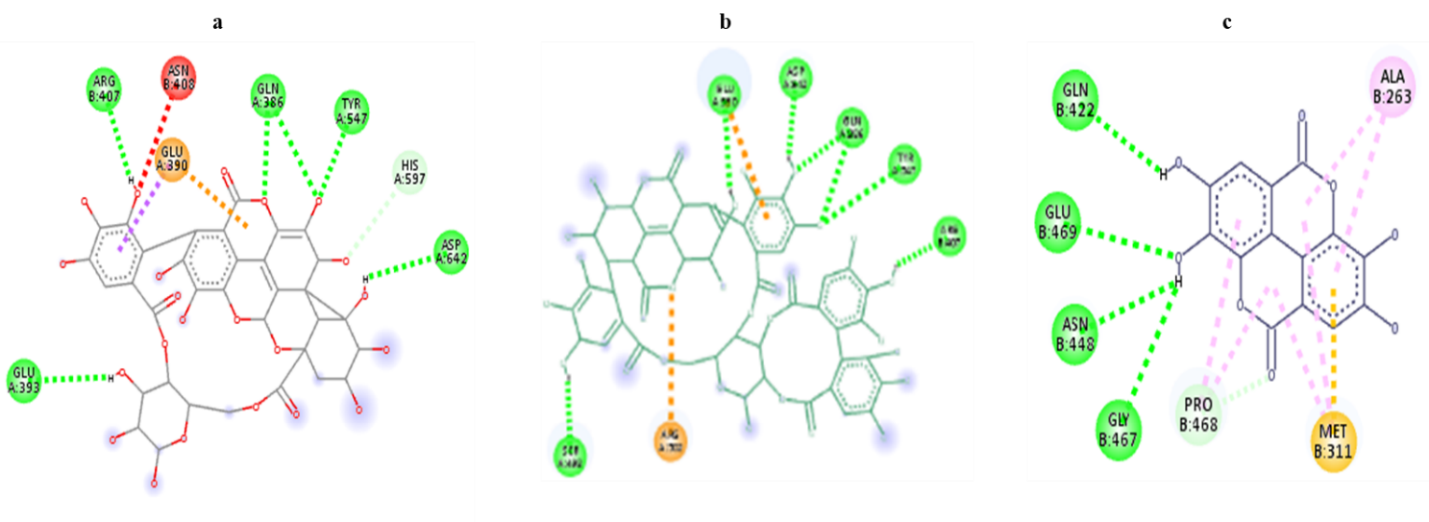


**Supplementary Figure 1.** 2D interactions of GFAT with punicalin (a), punicalagin (b) and ellagic acid (c). The 2D ligand interactions are depicted with pi-pi: green line, hydrogen bond: violet arrow line, 3D ligand interactions depicted hydrogen bond: purple dotted line, pi-pi: doted skyline.


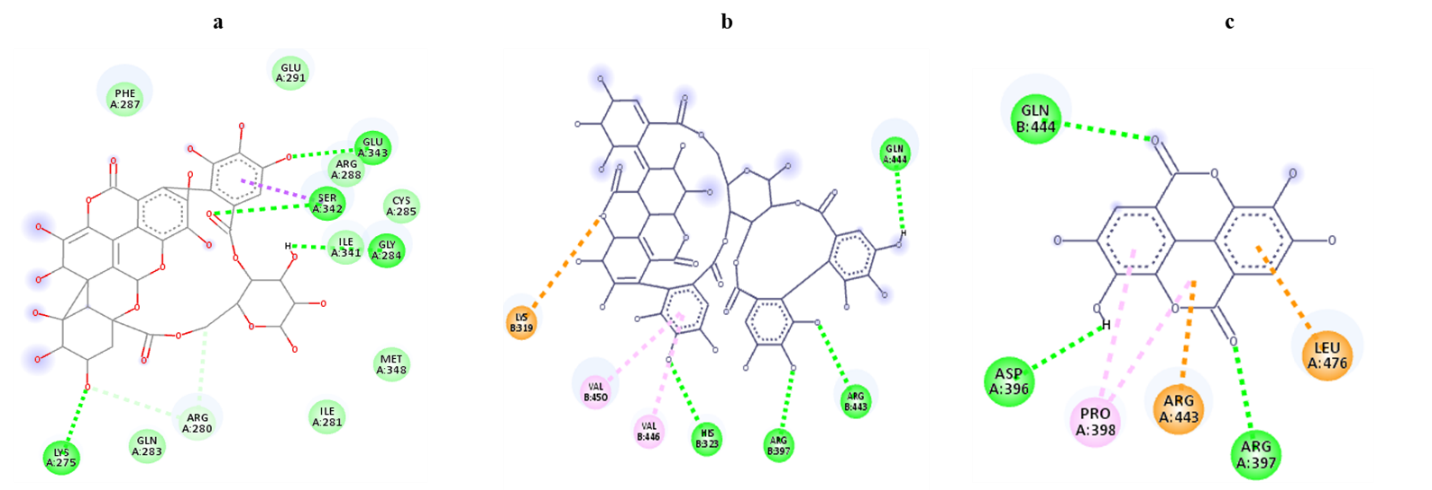


**Supplementary Figure 2.** 2D interactions of PPAR-ᵞ with punicalin (a), punicalagin (b) and ellagic acid (c). The 2D ligand interactions are depicted with pi-pi: green line, hydrogen bond: violet arrow line, 3D ligand interactions depicted hydrogen bond: purple dotted line, pi-pi: doted skyline.


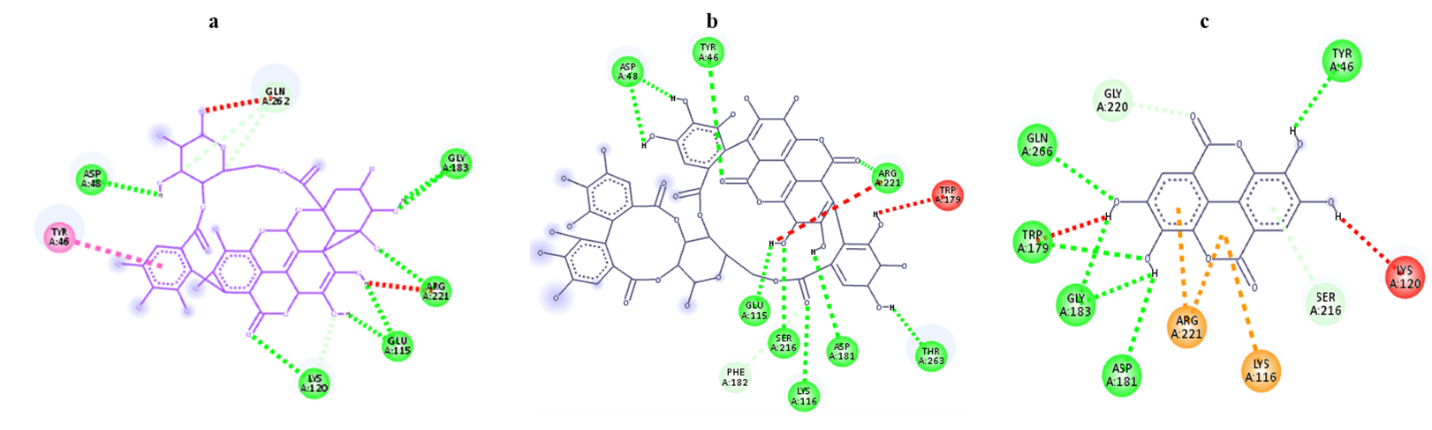


**Supplementary Figure 3.** 2D interactions of PTP1β with punicalin (a), punicalagin (b) and ellagic acid (c).The 2D ligand interactions are depicted with pi-pi: green line, hydrogen bond: violet arrow line, 3D ligand interactions depicted hydrogen bond: purple dotted line, pi-pi: doted skyline.


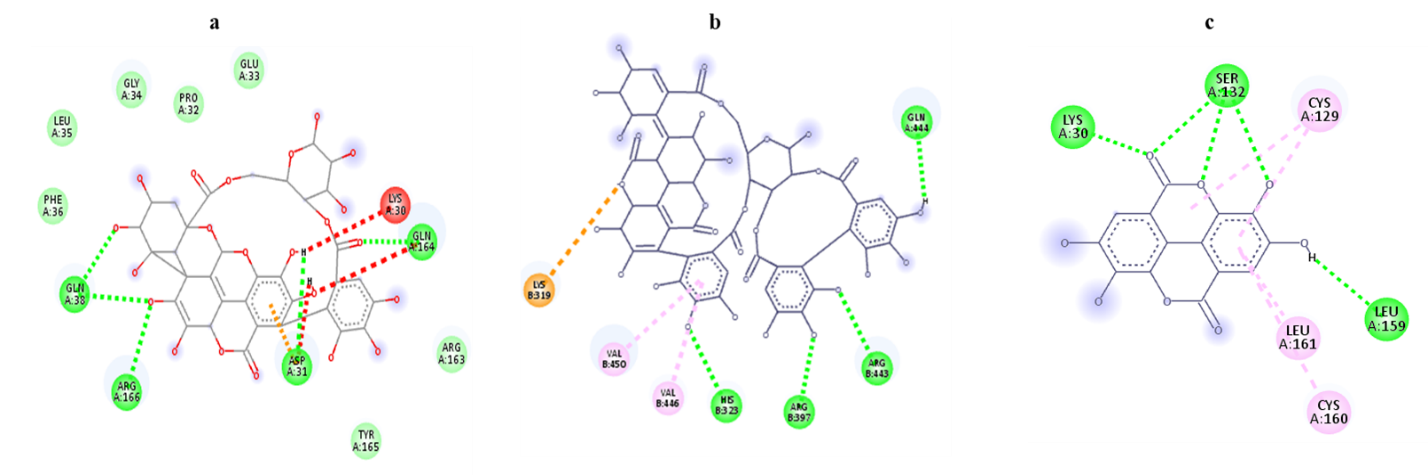


**Supplementary Figure 4.** 2D interactions of RBP4 with punicalin (a), punicalagin (b) and ellagic acid (c)).The 2D ligand interactions are depicted with pi-pi: green line, hydrogen bond: violet arrow line, 3D ligand interactions depicted hydrogen bond: purple dotted line, pi-pi: doted skyline.


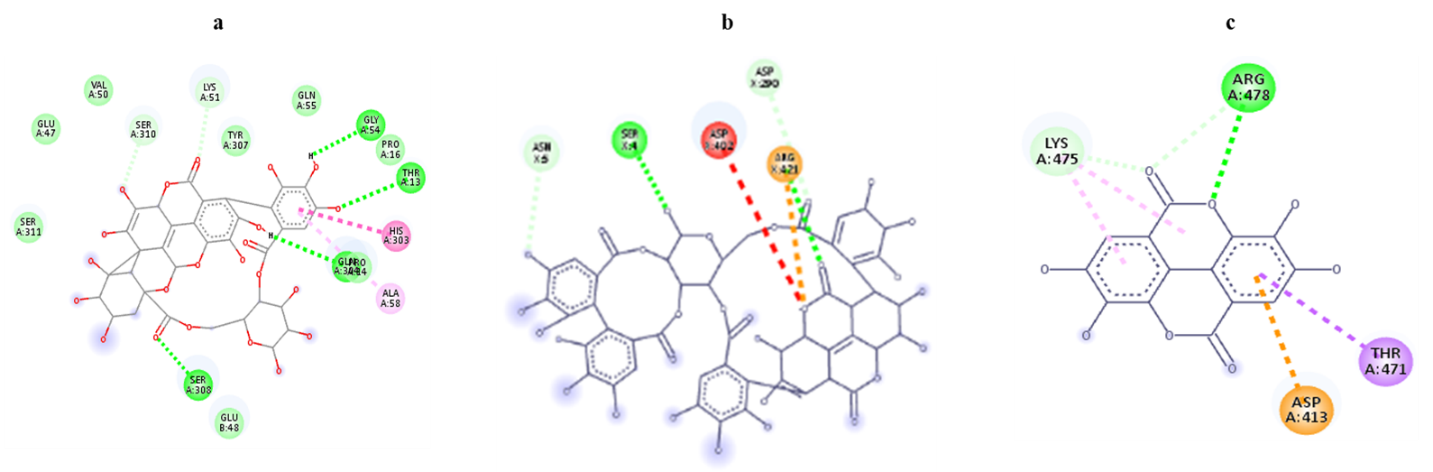


**Supplementary Figure 5.** 2D interactions of GCK with punicalin (a), punicalagin (b) and ellagic acid (c)).The 2D ligand interactions are depicted with pi-pi: green line, hydrogen bond: violet arrow line, 3D ligand interactions depicted hydrogen bond: purple dotted line, pi-pi: doted skyline.


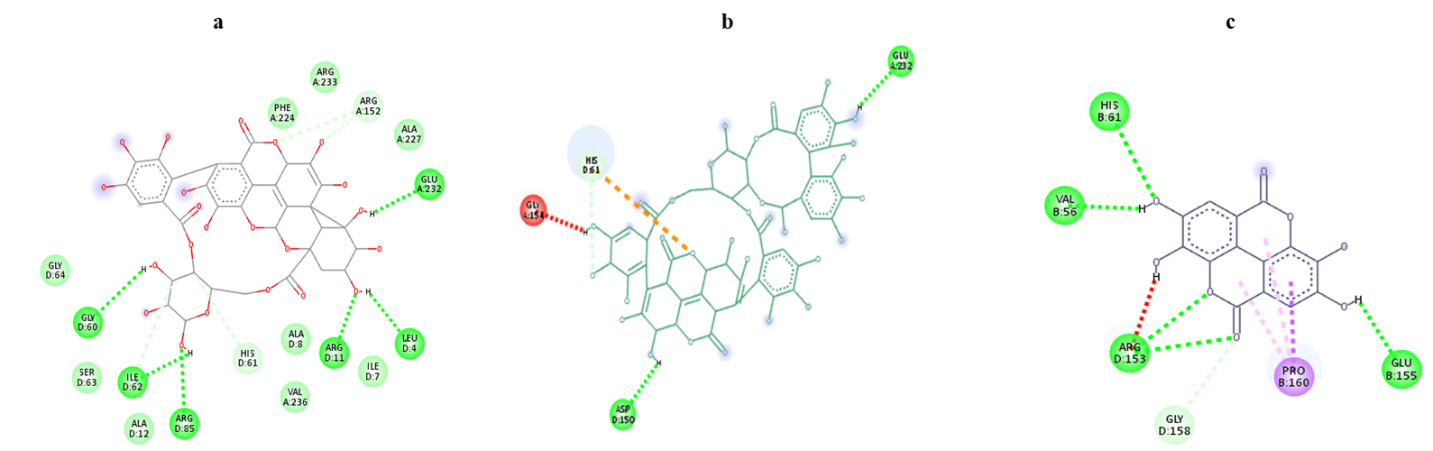


**Supplementary Figure 6.** 2D interactions of AQP-2 with punicalin (a), punicalagin (b) and ellagic acid (c)).The 2D ligand interactions are depicted with pi-pi: green line, hydrogen bond: violet arrow line, 3D ligand interactions depicted hydrogen bond: purple dotted line, pi-pi: doted skyline.


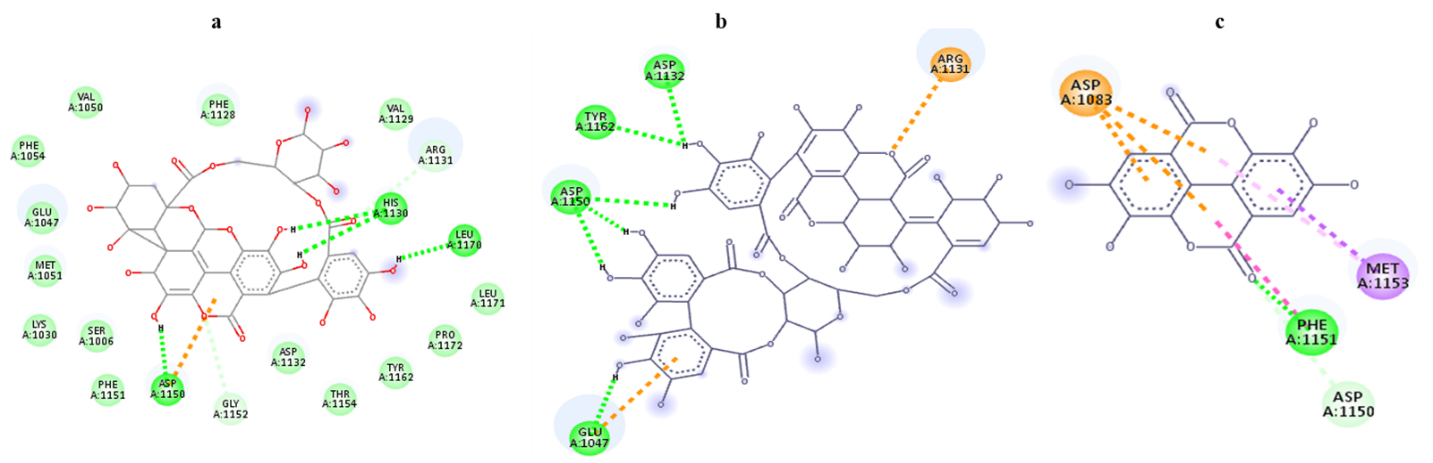


**Supplementary Figure 7.** 2D interactions of TK-IR with punicalin (a), punicalagin (b) and ellagic acid (c)).The 2D ligand interactions are depicted with pi-pi: green line, hydrogen bond: violet arrow line, 3D ligand interactions depicted hydrogen bond: purple dotted line, pi-pi: doted skyline.


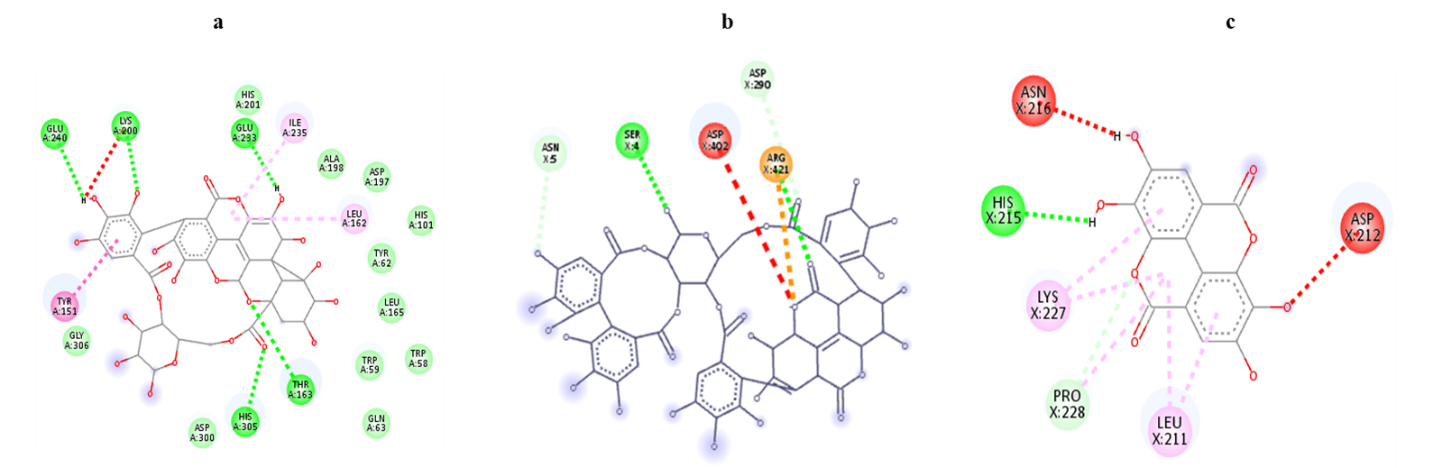


**Supplementary Figure 8.** 2D interactions of α-amylase with punicalin (a), punicalagin (b) and ellagic acid (c)).The 2D ligand interactions are depicted with pi-pi: green line, hydrogen bond: violet arrow line, 3D ligand interactions depicted hydrogen bond: purple dotted line, pi-pi: doted skyline.


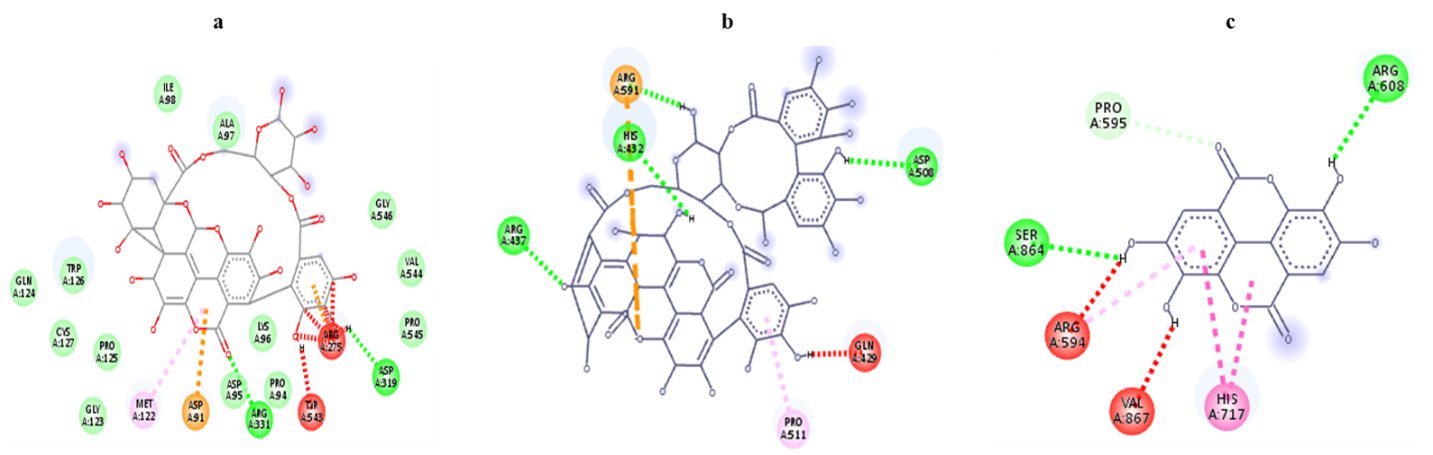


**Supplementary Figure 9.** 2D interactions of α-glucosidase with punicalin (a), punicalagin (b) and ellagic acid (c)).The 2D ligand interactions are depicted with pi-pi: green line, hydrogen bond: violet arrow line, 3D ligand interactions depicted hydrogen bond: purple dotted line, pi-pi: doted skyline.
